# Comparative Analysis of End Point Enzymatic Digests of Arabino-Xylan Isolated from Switchgrass (*Panicum virgatum* L) of Varying Maturities using LC-MS*^n ^*^†^

**DOI:** 10.3390/metabo2040959

**Published:** 2012-11-19

**Authors:** Michael J. Bowman, Bruce S. Dien, Patricia J. O’Bryan, Gautam Sarath, Michael A. Cotta

**Affiliations:** 1 U.S. Department of Agriculture, Agricultural Research Service, National Center for Agricultural Utilization Research, Bioenergy Research Unit, 1815 N. University Street, Peoria, IL 61604, USA; Email: Bruce.Dien@ars.usda.gov (B.S.D.); Pat.OBryan@ars.usda.gov (P.J.O.); Mike.Cotta@ars.usda.gov (M.A.C.); 2 U.S. Department of Agriculture, Agricultural Research Service, Central-East Regional Biomass Center, 137 Keim Hall, East Campus, UNL, Lincoln, NE 68583, USA; Email: Gautam.Sarath@ars.usda.gov (G.S.)

**Keywords:** switchgrass, xylan, xylooligosaccharides, LC-MS*^n^*, biofuel

## Abstract

Switchgrass (*Panicum virgatum* L., SG) is a perennial grass presently used for forage and being developed as a bioenergy crop for conversion of cell wall carbohydrates to biofuels. Up to 50% of the cell wall associated carbohydrates are xylan. SG was analyzed for xylan structural features at variable harvest maturities. Xylan from each of three maturities was isolated using classical alkaline extraction to yield fractions (Xyl A and B) with varying compositional ratios. The Xyl B fraction was observed to decrease with plant age. Xylan samples were subsequently prepared for structure analysis by digesting with pure endo-xylanase, which preserved side-groups, or a commercial carbohydrase preparation favored for biomass conversion work. Enzymatic digestion products were successfully permethylated and analyzed by reverse-phase liquid chromatography with mass spectrometric detection (RP-HPLC-MS*^n^*). This method is advantageous compared to prior work on plant biomass because it avoids isolation of individual arabinoxylan oligomers. The use of RP-HPLC- MS*^n^* differentiated 14 structural oligosaccharides (d.p. 3–9) from the monocomponent enzyme digestion and nine oligosaccharide structures (d.p. 3–9) from hydrolysis with a cellulase enzyme cocktail. The distribution of arabinoxylan oligomers varied depending upon the enzyme(s) applied but did not vary with harvest maturity.

## 1. Introduction

Switchgrass (*Panicum virgatum* L., SG) is a warm season perennial grass that is native to the North American prairies. Because of its high production yield and other favorable agronomic traits it is grown for forage production and is being developed as a bioenergy crop [1]. Biochemical conversion of switchgrass biomass into renewable biofuels and chemicals begins with the extraction of carbohydrates using a combination of thermal, chemical, and enzymatic processing steps. The majority of the carbohydrates are contained in the primary and secondary plant cell walls. The primary cell wall of grasses are composed of polysaccharides cellulose (20%–30%), xylan (20%–40%), and pectin (5%) with a minor polyaromatic lignin component; the secondary cell walls have an increased quantity of cellulose (35%–45%), xylan (40%–50%), and lignin (20%), with a decreased pectin content (0.1%) [2]. The high content of xylan within the grass cell wall material makes it an important factor in the conversion process; as both an impedant to efficient cellulose conversion [3,4] and as a source of pentose sugars [5]. Grass xylan is a heterogeneous polymer consisting of repeating (1→4)-β-linked xylose residues with variability in acetyl or arabinose substitutions at the 2-*O* and/or 3-*O* positions of backbone xyloses. Further complexity of the chains can arise from additional substitutions (hexose, pentose, acetyl, and/or phenolics) on the arabinose side chains [6,7]. 

Lignocellulose is recalcitrant to enzymes and, therefore, pretreatment is required to open up the cell wall structure. Numerous pretreatment strategies have been proposed in the literature [8]. The xylan structure affects the processing of biomass at the pretreatment step. For example, when biomass is pretreated with a mineral acid catalyst at relatively high temperatures (e.g., 160–200 °C); xylan hydrolysis displays bimodal kinetics with fast and slow (20%–35% of total xylan) reacting fractions [9]. The slow kinetic fraction partially sets the minimal pretreatment severity. Following pretreatment, the carbohydrate polymers are hydrolyzed to sugars with enzymes either prior to or simultaneously with fermentation. Knowledge of the xylan structure can be directly used in formulating the xylanase cocktail so as to obtain the highest yield with the lowest number of enzymes. Partially digested xylan is a source of fermentation loss and interferes with cellulose hydrolysis, presumably by competitive inhibition of cellulases [3].

Analyses of intact xylan samples are infeasible because of their size and heterogeneity. Therefore xylan samples are hydrolyzed with enzymes (or acid catalysts) into arabino-xylooligosaccharides (AXOs). The resulting oligomers can be fractionated by large-scale separation where oligosaccharide structural assignments are made by linkage analysis and nuclear magnetic resonance (NMR) and isolated products used as standards for high performance anion exchange chromatography with pulsed amperometric detection (HPAEC-PAD) analysis for routine measurement [10,11,12,13,14,15]. Due to the complexity of AXO products, alternative chromatographic techniques are necessary, as incomplete separation has been shown for isomeric arabino-oligomers (*i.e.* oligomers from poly-arabinose) using HPAEC-PAD [16]. Several reports employing the isolation and characterization strategy also use infusion electrospray ionization mass spectrometry to further characterize the isolated materials either as native oligosaccharides [13,17,18] or as permethylated derivatives [17,19,20,21]. Prior infusion mass spectrometry (MS) reports are largely limited to studies of pericarp arabinoxylan as a source of branched AXOs [13,17,18,19,22,23] because it is convenient to obtain; one prior study has reported on endo-xylanase digested SG cell wall AXOs [20]. These prior studies are further limited to those isolated AXOs that are typically the most abundant oligosaccharide products of the digestion and do not consider low abundance sequences.

The isolation of AXO products is a time consuming process that is not conducive to identifying low abundance sequences. Mass spectrometry is a sensitive analytical technique that can provide structural information about carbohydrate analytes by variations in ionization [24], fragmentation chemistries [24], and derivatization [24,25], that has been applied to characterize a variety of plant cell wall polysaccharides [26]. As a complement to a purification and characterization strategy of the products, a permethylation, reverse-phase high-performance liquid chromatography multiple stage mass spectrometry (RP-HPLC-MS*^n^*) strategy is proposed herein. RP-HPLC-MS*^n^* can be an informative technique for the identification, structural assignment, and relative quantification of the products of endo-xylanase treatment of arabinoxylans from food or biomass sources. Hydrophilic-interaction chromatography mass spectrometry LC-MS/MS has been used to identify reducing-end labeled AXOs [22,23,27,28]; however the isobaric constituents of xylose and arabinose have identical neutral losses in the resultant glycosidic fragment ions that prevent unambiguous MS*^n^* analysis. 

This study had two goals. The first was to enhance current analytical methods for isolated xylans from grasses to allow for rapid structure determinations. The second was to apply this method to determine if SG alters its xylan structure as the plant grows. For the later purpose, SG samples were analyzed from plants harvested at early, middle, and late maturities. Harvest maturity affects both forage digestion and biofuel conversion kinetics and yields [29,30,31]. The isolated xylans from these samples were enzymatically depolymerized, reduced, permethylated, and analyzed by C_18_-RP-HPLC-based liquid chromatography with mass spectrometry (LC-MS*^n^*), Permethylation facilitates structural assignments at branch points because the glycosidic cleavage fragments contain an identifiable mass shift that is not present in native structures. C_18_-RP-HPLC-based LC-MS*^n^* was used to provide a more complete picture of the AXO structures compared to HPAEC-PAD, because data can simultaneously be collected on the degree of polymerization (d.p.) and fragmentation ions that identify structural differences. In addition, the increased sensitivity of mass spectrometry permitted identification of low abundance components within the digestion mixture. 

## 2. Results and Discussion

### 2.1. Xylan Isolation

SG samples were obtained at different harvest dates because it is well known that plant maturity affects biomass quality [29,30,31]. Xylan was isolated from SG at three harvest maturities, which were pre-boot (MPV1), where leaf is in greater quantity than stems; anthesis (MPV2), where leaf and stem are approximately equal; and post-frost (MPV3), where stem is the most prevalent. These specific SG samples had been characterized for conversion yields by this laboratory and greater maturity was determined to lower (on a specific carbohydrate basis) enzymatic release of free sugars following dilute acid treatment [30] and ethanol conversion after pretreatment with ammonium hydroxide [29]. While the observed differences are likely the result of multiple factors, xylan is known to interfere with cellulose accessibility [4], to inhibit cellulolytic enzymes [3] and to alter the kinetics of acid catalyzed depolymerization [9].

Triplicate samples of each maturity were delignified with chlorous acid. Xylan was then separated from cellulose by alkaline extraction, prior to xylan fractionation, based on low aqueous solubility (xylan A) and subsequent precipitation by addition of organic solvent (xylan B), using well-established techniques [32]. The low aqueous solubility of xylan A is due to a lower arabinose substitution, therefore, xylan A may have a greater effect on cellulose conversion because long, unsubstituted stretches of xylan have been proposed to bind more strongly to cellulose [4]. In contrast, the increased substitution of xylan B, which makes it the more water-soluble fraction, was expected to have more diverse structures because of the increased arabinose substitutions. The influence of ester bond groups (e.g., acetyl- and feruloyl-esters) went unexamined because the alkaline extraction cleaves these bonds. Slight changes in relative amounts of fraction less-substituted, water-insoluble xylan A was noted for the early *versus* the later maturities samples. The preboot SG (MPV1) contained 7.25 ± 0.34% (g/g dry mass) whereas 7.75 ± 1.10% and 7.71 ± 0.93% dry mass were measured for MPV2 and MPV3, respectively. In contrast, the preboot SG (MPV1) contained a higher percentage of xylan B (23.2 ± 1.7%) than MPV2 (20.2 ± 1.9%) and MPV3 (17.2 ± 2.3%). Overall, MPV1 was enriched with xylan while MPV2 and MPV3 contained greater amounts of cellulose and lignin. 

The monosaccharide compositions of the each isolated holocellulose and xylan fraction were determined by complete acid hydrolysis using the National Renewable Energy Laboratory (NREL) protocol 510-42618 [33]. The xylose to arabinose (Xyl:Ara) ratio is nearly constant for xylan A fractions (X:A ratios of 10.4 ± 0.6, 10.3 ± 0.8, 10.6 ± 0.6), however the xyl:ara ratio in xylan B is lowest for MPV1 SG (X:A ratios of 4.4 ± 0.4, 6.7 ± 0.7, 6.5 ± 0.2), thereby showing greater arabinose substitution at early maturity. It is notable that the mass fractions of xylan A and xylan B present in SG are similar to those previously reported for reed canary grass [34]. 

### 2.2. Enzymatic Depolymerization

Xylan needs to be depolymerized into oligomers prior to structural analysis. To this end, isolated switchgrass xylan fractions were treated with endo-xylanase (E.C.3.2.1.8) from *T. viride*, a commercially available and commonly used enzyme for structural analysis [10,11,12,13,15,20], to generate a limited number of end point arabino-xylooligosaccharide products. This endo-xylanase is a member of the glycohydrolase (GH) family 11, containing a characteristic “thumb” loop as part of the binding pocket that provides high substrate selectivity for xylan. Due to the high selectivity of GH11 endo-xylanases for unsubstituted regions of the xylan chain, hydrolysis leaves side groups intact. Additionally, GH11 xylanases have longer binding pockets than xylanase GH families 5, 8, and 10 (GH xylanases are extensively reviewed in [35,36,37]) leading to larger oligosaccharide products. Previous reports characterized the specificity of *T. viride* xylanase to generate primarily branched arabino-xylooligosaccharides, xylose, and xylobiose from wheat arabinoxylan [12,13]; therefore, wheat arabinoxylan was included as a control for enzyme digestion, labeling, and chromatography. To assess the activity of *T. viride* endo-xylanase on SG xylan a preliminary experiment was performed, xylan A and xylan B of MPV-2 were incubated with enzyme, as described in Section 3.2., with aliquots (1, 2, 8, 12, 16, 24, and 48 h) analyzed by HPAEC-PAD. Chromatographic peaks changed in signal intensity from 1 to 12 h, after 16 h the product profiles remained constant. After 16 h, additional enzyme was added to confirm that inactivation of enzyme was not responsible for the observed end of digestion. The temperature and buffer used were consistent with temperature and pH optima for the endo-xylanase, as reported by the manufacturer. Enzyme loading was in excess of the calculated amount needed to hydrolyze the xylan sample. 

Treatment of the xylan samples was also generated using a commercial *T. reesei* cellulase preparation commonly used for biomass conversion in the literature. While it may seem odd to apply a cellulase mixture for xylan hydrolysis, commercial cellulase solutions are prepared from fungal cultures and contain a wide spectrum of biomass related hydrolytic activities including: two cellobiohydrolases (E.C.3.2.1.91), five endo-glucanases (E.C.3.2.1.4), two β-glucosidases (E.C.3.2.1.21), four xylanases (E.C.3.2.1.8), a mannanase (E.C.3.2.1.78), a β-xylosidase (E.C.3.2.1.37), a β-mannosidase (E.C.3.2.1.25), three α-arabinofuranosidases (E.C.3.2.1.55), an α-glucouronidase (E.C.3.2.1.139), and an α-galactosidase (E.C.3.2.1.22). The xylanases of *T. reesei* belong to GH families 5 (XynIV) [38], 10 (XynIII), and 11 (XynI and XynII) [39]. While the *T. reesei* cellulase preparation represents a mixture of activities, its prevalent use in biomass conversion was expected to provide insight into the depolymerization of xylan. Overall xylanase and xylan debranching activities on model substrates for the specific cellulase used here, Celluclast, had been characterized earlier [40,41]. For parallel processing of samples, the *T. reesei* enzyme cocktail loading was kept consistent with *T. viride* endo-xylanase (*i.e.*, 2U xylanase) for xylanase activity and the pH, temperature, and buffer conditions were consistent with those reported for enzyme activity assays [41]. At this enzyme loading and reaction time auxiliary enzyme (*i.e.* α-arabinofuranosidase) activity was not expected to be limiting. 

Both xylan fractions (A and B) from each SG maturity (MPV-1, MPV-2, MPV-3) were independently digested and profiled. Xylan samples were normalized for xylose content to cancel out effects related to differences in relative xylan contents at different harvest maturities [30]. It was expected that changes in oligosaccharide structure would result in the appearance of new signals in these xylose-normalized samples. The endpoint for enzymatic digestion, its reproducibility, and preliminary enumeration of products (nine PAD peaks for *T. viride* endo-xylanase digestion and six for *T. reesei* enzyme cocktail digestion; supplemental Figure 1 were determined by HPAEC-PAD analysis. 

**Figure 1:**
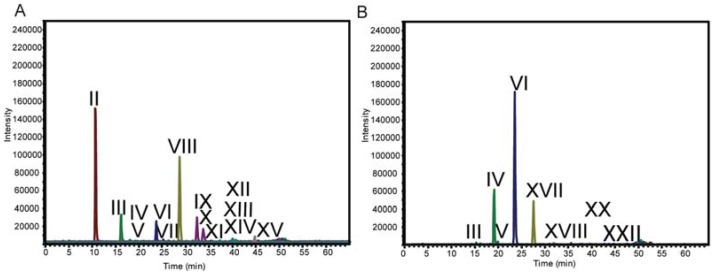
C_18_-LC-MS of enzymatically depolymerized MPV-2 xylan B (**a**) extracted-ion chromatograms (EICS) of oligosaccharide products of *T. viride* endo-xylanase enzymatic end point treatment of switchgrass xylan (d.p.2-d.p.8). (**b**) EICs of oligosaccharide products of Celluclast end point treatment of switchgrass xylan (d.p.2-d.p.8). EICs corresponding to: brown (Pent)_2 _PM (*m/z* 405); green (Pent)_3 _PM (*m/z* 535); blue (Pent)_4 _PM (*m/z* 725); gold (Pent)_5_PM (*m/z* 885); purple (Pent)_6 _PM (*m/z* 1045); aqua (Pent)_7 _PM (*m/z* 1205); grey (Pent)_8_PM (*m/z* 1365).

### 2.3. LC-MS

Permethylation of AXO samples enhanced chromatographic separation, increased signal strength, and generated more informative fragmentation patterns, in a similar manner as reviewed for other carbohydrates [24,25]. AXO structures have been determined by infusion MS*^n^* of enriched or purified permethylated AXOs [17,19,20]; however, LC-MS*^n^* has not been applied to permethylated biomass AXOs. Permethylation allows for semi-quantitative analysis due to the normalizing effect of permethylation on ionization efficiencies [42] and allows for the use of chromatographic matrices that are capable of isomeric separations of permethylated oligosaccharides from other sources [42,43,44]. To account for losses during the liquid-liquid extraction step of the permethylation protocol, maltotetraose was added prior to reduction and used as an internal standard. 

**Figure 2:**
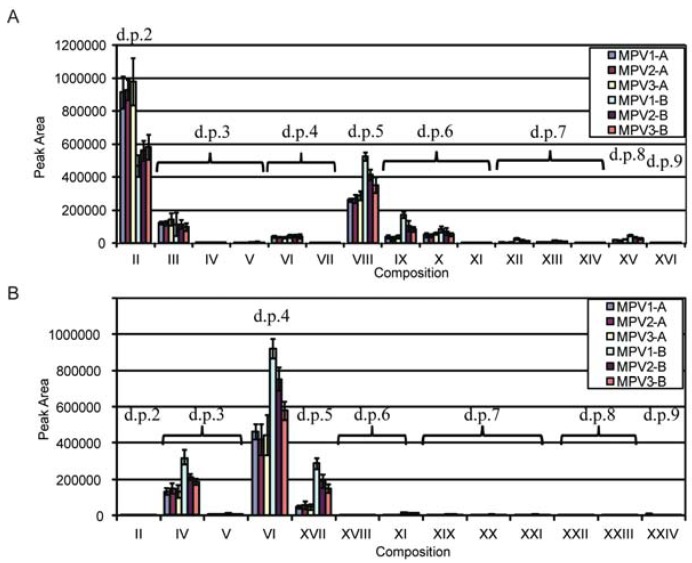
(**a**) Graphical representation of differences in the quantities of oligosaccharide products of switchgrass (SG) structural xylan from each maturity and xylan class from *T. viride* endo-xylanase enzymatic end-point digestion. (**b**) Graphical representation of differences in the quantities of oligosaccharide products of SG structural xylan from each maturity and xylan class from Celluclast end-point digestion.

**Figure 3:**
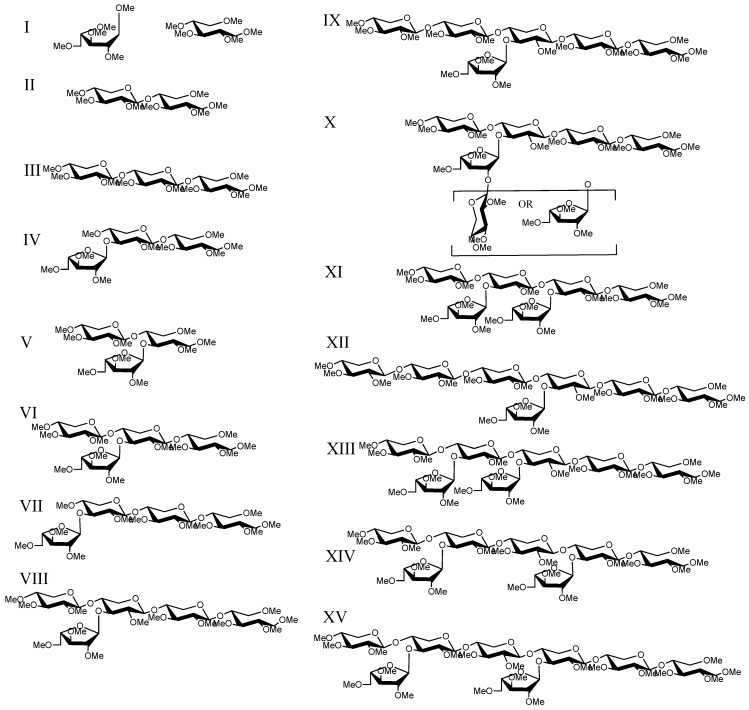
Structures present in *T. viride* endo-xylanase digested SG xylan.

Analysis by reverse-phase C_18 _HPLC-MS*^n^* in the positive mode provided strong singly-charged ([M+Na]^+^) peaks corresponding to the masses for the reduced, permethylated oligosaccharides (Table 1); no higher charge states were observed. Using Xyl_2_-Xyl_6_ standards and structural variations determined by LC-MS*^n^* (*vida infra*), the retention time trend for oligosaccharides increased with branching (linear, single pentose branch, double pentose branch). There were differences in the oligosaccharide product profiles in the extracted-ion chromatograms (EICs) from the two digestions (*T. viride* endo-xylanase *vs. T. reesei* enzyme cocktail; Figure 1 and Figure S2) as expected because of quantitative and qualitative differences between the enzyme solutions. Just as notable, there were no additional structures observed with changes in maturity or between the differentially substituted xylan A and xylan B fractions (14 for *T. viride* endo-xylanase, and nine for *T. reesei* enzyme cocktail, Figure 1 and Figure 2). The oligosaccharides range from d.p. 2 to d.p. 9, each spaced by 160 daltons corresponding to the mass of a permethylated pentose; no hexose or hexuronic acid containing oligosaccharides were observed. Structures II and III were assigned by comparison of retention times and MS^2^ fragmentation patterns to linear Xyl_2_ and Xyl_3_ standards (Figure 3). Unlabeled EIC peaks in Figure 1 represent non-oligosaccharide coincidental mass peaks that have tandem mass spectra inconsistent with permethylated oligosaccharides.

**Table 1:** Characteristics of Pentose Oligomers analyzed in this study.^a^

Structure	d.p.^a^ (RT, min)	Underivatized Molecular Formula	Reduced, Permethylated *m/z* (M+Na)^+^	Assignment by	Present in	Additional Information/References
I	1	C_5_H_12_O_5_	245.1	Standards	*T. reesei* digestion	
II	2 (10.9)	C_10_H_20_O_9_	405.2	Standard	*T. viride* digestion	
III	3 (16.3)	C_15_H_28_O_13_	565.3	Standard	*T. viride* digestion	
IV	3 (19.2)	C_15_H_28_O_13_	565.3	MS *^n^* Fragmentation	*T. viride* and *T. reesei* digestion	Linear sequence that does not correlate with Xyl_3_ standard
V	3 (19.8)	C_15_H_28_O_13_	565.3	MS *^n^* Fragmentation	*T. viride* and *T. reesei* digestion	
VI	4 (23.5)	C_20_H_36_O_17_	725.4	MS *^n^* Fragmentation	*T. viride* and *T. reesei* digestion	[12,13,18,45]
VII	4 (25.1)	C_20_H_36_O_17_	725.4	MS *^n^* Fragmentation	*T. viride* digestion	Linear sequence that does not correlate with Xyl_4_ standard
VIII	5 (28.5)	C_25_H_44_O_21_	885.4	MS *^n^* Fragmentation	*T. viride* digestion	[12,13,18,20,45]
IX	6 (32.4)	C_30_H_52_O_25_	1045.5	MS *^n^* Fragmentation	*T. viride* digestion	Tentative assignment may be X
X	6 (33.6)	C_30_H_52_O_25_	1045.5	MS *^n^* Fragmentation	*T. viride* digestion	[20] Peak may be IX
XI	6 (35.5)	C_30_H_52_O_25_	1045.5	MS *^n^* Fragmentation	*T. viride* and *T. reesei* digestion	[12,18]
XII	7 (37.5)	C_35_H_60_O_29_	1205.6	MS *^n^* Fragmentation	*T. viride* digestion	
XIII	7 (39.9)	C_35_H_60_O_29_	1205.6	MS *^n^* Fragmentation	*T. viride* digestion	
XIV	7 (40.7)	C_35_H_60_O_29_	1205.6	MS *^n^* Fragmentation	*T. viride* digestion	
XV	8 (44.9)	C_40_H_68_O_33_	1365.7	MS *^n^* Fragmentation	*T. viride* digestion	[18]
XVI	9 (48.6)	C_45_H_76_O_37_	1525.7	Retention Time	*T. viride* digestion	
XVII	5 (27.5)	C_25_H_44_O_21_	885.4	MS *^n^* Fragmentation	*T. reesei* digestion	[27]
XVIII	6 (32.8)	C_30_H_52_O_25_	1045.5	MS *^n^* Fragmentation	*T. reesei* digestion	
XIX	7 (38.9)	C_35_H_60_O_29_	1205.6	MS *^n^* Fragmentation	*T. reesei* digestion	
XX	7 (40.9)	C_35_H_60_O_29_	1205.6	MS *^n^* Fragmentation	*T. reesei* digestion	
XXI	7 (42.9)	C_35_H_60_O_29_	1205.6	MS *^n^* Fragmentation	*T. reesei* digestion	
XXII	8 (43.8)	C_40_H_68_O_33_	1365.7	Retention Time	*T. reesei* digestion	
XXIII	8 (44.8)	C_40_H_68_O_33_	1365.7	Retention Time	*T. reesei* digestion	
XXIV	9 (47.5)	C_45_H_76_O_37_	1525.7	Retention Time	*T. reesei* digestion	
(Glc)_4_-Internal Standard		C_24_H_44_O_21_	901.5			

^a ^d.p. represents number of pentose units

Within the *T. viride* endo-xylanase-digested xylan set, SG xylan A showed an increased presence of Structures II and III, as expected because xylan A is less substituted and more susceptible to endo-xylanase enzymes for the generation of linear xylo-oligosaccharides. Comparison within the *T. viride* endo-xylanase-digested xylan A set showed that EIC peak areas for all other measurable oligosaccharide signals did not differ with maturity, which is reflective of the reduced substitution and similarity between Xyl:Ara ratios for each maturity. This indicates that change in the amount of xylan A is the greatest source of variability with maturity. Interestingly, no additional peaks from *T. viride* endo-xylanase-digested xylan B fractions, compared to *T. viride* endo-xylanase -digested xylan A, were detected by C_18_-LC-MS, where they may have been expected due to higher arabinose substitution. The more substituted xylan B had a more divergent Xyl:Ara ratio in early maturity that increased (*i.e.*, became less substituted) with maturity. The LC-MS data are reflective of this trend as substituted oligosaccharides had lower signals with increased maturity, and signals for unsubstituted oligosaccharides II and III increased. Furthermore, the *T. viride* endo-xylanase-digested xylan B MS data showed higher oligosaccharide signals compared to xylan A. This was not unexpected as the higher arabinose substitution of the xylan B fractions was not the result of new structures present in xylan B, but rather an increase in the prevalence of substituted structures. Xylan B has lower measurable quantities of linear oligosaccharides II and III compared to xylan A, also consistent with increased arabinose substitution. Interestingly, two d.p. 6 oligosaccharides, with nearly equivalent signals (IX and X), and a third low-abundance d.p. 6 (XI) were observed in both SG xylan A and B samples, in contrast to the previous report of a single hexasaccharide from Alamo SG containing an α-ara-(1→2-)-α-ara (1→3-) disaccharide side-group [20], when fractionated by only size-exclusion chromatography. 

In contrast, Celluclast digestion of xylan fractions A and B were more complete (*i.e.*, a greater quantity of monosaccharide products were measured by HPAEC-PAD, supplemental Figure 1), which was likely due to the presence of auxiliary debranching enzymes [40] providing greater substrate access for endo-xylanases, while GH10 (XynIII) and GH5 (XynIV) further hydrolyzed the AXO products of GH11 enzymes (XynI and XynII) [36,37]. Consistent with what was observed for the *T. viride* endo-xylanase-digestion of xylan fractions, oligosaccharide peaks were the same for xylan A and B, xylan A digestion profiles were similar across maturities, and the substituted oligosaccharide signals for samples of xylan B decreased with greater maturity. However, AXOs from *T. viride* endo-xylanase and *T. reesei* enzyme cocktail digestions had different retention times and fragmentation patterns indicating the presence of isomers resulting from the altered substrate recognition of the two xylanase sources. This demonstrates that reverse-phase- liquid chromatography with mass spectrometry (RP-LC-MS) is suitable for differentiating isomeric AXOs from different enzymatic treatments. 

### 2.4. LC-MS^2^

Several reports characterize the end-point products of *T. viride* endo-xylanase treatment of xylan using separation techniques (gel-filtration and/or semi-preparative HPAEC) to isolate oligosaccharide products from various sources [13,17,18,20,45]. This method generates AXOs that can be readily interrogated to determine structural features by permethylation and MS*^n^*. The use of this monocomponent enzyme was expected to generate AXOs from SG xylan of similar structural character to those previously reported for wheat pericarp xylan due to the enzyme’s substrate selectivity. With the exception that grass cell wall xylan is expected to posses only α-(1→3)-arabinose branches, as this is the major linkage reported for SG [20] and other grasses [7]. MS*^n^* is capable of identifying the branch point and the linkage position of pentose substitutions, but differentiating between arabinose and xylose substitutions relies on differences between the furanose for arabinose and pyranose for xylose forms of the pentoses. Therefore, all structures are proposed based on the previously reported prevalence of α-(1→3)-arabinose branches and disaccharide branches as either (1→2)-α-ara-(1→3)-α-ara or (1→2)-β-xyl-(1→3)-α-ara for grasses [7,20]. 

**Figure 4:**
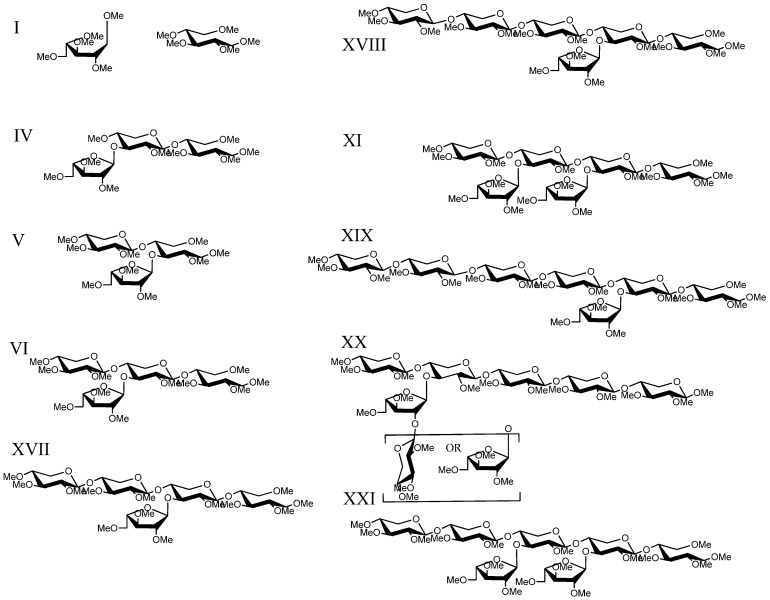
Structures present in Celluclast digested SG xylan.

Using LC-MS*^n^*, several structures (II, VI, VIII, X, XIII, XV, Figure 3 and Figure 4) can be assigned for analyte peaks that are consistent with the major *T. viride* endo-xylanase end-point digestion products previously reported from biomass and pericarp sources [13,17,18,20]. The presence of these end-point digestion products reflects the consistency of the product oligosaccharide selectivity of the *T. viride* endo-xylanase treatment among xylan sources. Permethylation of the analyte facilitates structural identification, as the tandem mass spectrometric data from LC-MS^2^ are informative in the determination of branch points in the xylose backbone. Under the fragmentation conditions used, tandem spectral data are dominated by Y- and B-type single fragmentations (in accordance with the nomenclature of Domon and Costello [46]), with double B-/Y- and Y-/Y-fragmentation ions also present. 

**Figure 5:**
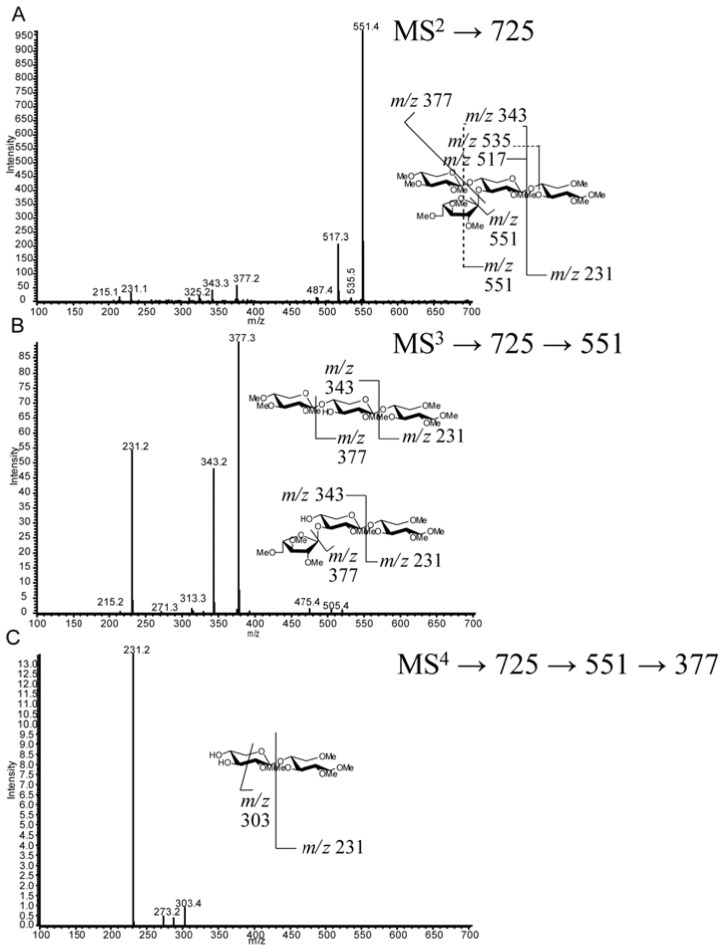
Mass spectrometry (MS*^n^*) fragmentation of permethylated oligosaccharide VI (RT 23.5 min) (**a**) MS^2^* m/z* 725. (**b**) MS^3^ fragmentation *m/z* 725→551. (**c**) MS^4^ fragmentation *m/z* 725→551→377.

For example, MS^2^ fragmentation of structure VI (a d.p.4; Figure 5a), present in both *T. viride* endo-xylanase and *T. reesei* enzyme cocktail digestion samples, yields an intense Y_2_ ion (*m/z* 551), a B_2_ ion (*m/z* 517), an internal B_2_/Y_2_ ion (*m/z* 343), and a Y_2_/Y_2α_ fragment (*m/z* 377) that gives strong evidence for a pentose branch being present on the internal xylose backbone (Figure 3 and Figure 4). This glycosidic bond fragmentation pattern was consistent for all oligosaccharide ions detected, the point of backbone substitution can be deduced, in cases of one-pentose-branch(es), by the absence of both Y- and complementary B-ion ions from a predicted linear sequence (*i.e.*, *m/z* 391 and *m/z* 357, Figure 5a). Due to the known branching of grass cell wall arabinoxylan it is possible to have a two-pentose branch ((1→2)-α-ara-(1→3)-α-ara or (1 →2)-β-xyl-(1→3)-α-ara) that may confound some structural analyses. Unfortunately, differentiation of structural possibilities cannot be made for the assignment of a two-unit branch (Structure X) *versus* a one-unit branch two units from the non-reducing end (Structure IX), as the distinct retention times and fragmentation patterns from the two peaks demonstrate that d.p. 6 isomers are present. Therefore, the assignments of IX and X are to be treated as tentative. Data collected from the *T. reesei* cellulase enzyme cocktail (Celluclast) provided MS^2^ fragmentation patterns that can be used to identify alternate sites of branching, as the selectivity of xylanase enzyme(s) gave an altered pattern of products (*i.e.*, *T. viride* favors the generation of oligosaccharides branched at the third residue from the reducing end (Figure 3), whereas *T. reesei* xylanases produce oligosaccharides branched at the second residue from the reducing end (Figure 4).) It should be noted that the enzyme cocktail may also have exo-enzymatic activities that clip monosaccharide units (xyl and ara) from the initial *T. reesei* endo-xylanase product oligosaccharides to smaller pieces; however the end-point products are constant with maturity and xylan type, showing the fidelity of the enzyme cocktail treatment. The *T. reesei* AXO products have direct significance to biomass conversion because of the prevalence of *T. reesei*-based enzyme cocktails for biomass saccharification. 

Due to the complexity and low signals of the largest oligosaccharides detected, structural assignments could not be made for Structures XVI, XXII, XXIII, and XXIV.

### 2.5. LC-MS^n^

In some cases MS^2^ was not sufficient for complete structural assignments; therefore, additional fragmentation data from the segmented MS*^n^* method was used. Within the appropriate retention time window for each d.p. oligomer, MS^2^ targeted the appropriate *m/z* for the AXO d.p. followed by an MS*^n^* tree that targeted ions in the following manner: In MS^3^ the Y- and B-ions corresponding to the loss of one pentose (Y_(d.p.-1)-14_; B_(d.p.-1)-14_), followed by subsequent MS*^n^* for fragmentations of pentose losses (Y_(d.p.-2)-14_; B_(d.p.-2)-14_); the isolation and fragmentation of double losses were also included (e.g. Y/Y_α(d.p.-2)-28_), as these represent the information-rich fragments that show where branching has been present. There were up to 13 MS*^n^* experiments within a time window, encompassing MS^2^ to MS^6^; however data from MS^5^ and MS^6^ were typically limited in information. The time required to complete the MS*^n ^*tree was limiting; therefore, a traditional 250 mm HPLC column was used to provide sufficient resolution of analytes while permitting the full MS*^n^* analyses of the targeted ions. Peak elution profiles under ultra-performance liquid chromatography (UPLC) conditions were insufficiently long to obtain detailed structural information from the MS*^n^* tree (data not shown); however UPLC may be used once detailed structures are determined and MS^2^ patterns are sufficient to confirm the identity of known analytes. MS^2^ data were ambiguous for the interpretation of smaller oligosaccharides (d.p. ≤ 3); however the use of linear standards (Xyl_2_-Xyl_6_) and MS*^n^* provided sufficient data for assignments of II and III, and excluded the presence of linear xylooligomers as d.p 4–6 products. *T. viride* endo-xylanase digestion produces a linear xylotriose (Xyl_3_, III) indicating that the number of xylose repeats between endo-xylanase product oligosaccharide structures within the xylan should be an odd number. Xyl_3_ has been reported as a product of *T. viride* endo-xylanase digestion [45], but primarily generates xylobiose as a product from Xyl_4_ and Xyl_6_ (data not shown), showing a strong preference for generation of xylobiose. In addition to chromatographic and mass spectrometric comparisons to known linear standards, assignments for additional d.p. 3 structures can be made based on MS*^n^* data. For example, structure V can be identified by the presence of the sole ion at *m/z* 217 in its MS^3^ spectrum (*m/z* 565→391) (Supplemental Figure 3), corresponding to a monosaccharide unit on the reducing end that had a branching pentose [19]. This low-abundance product may be the result of *T. reesei* GH5 (XynIV) hydrolysis, as a GH5 xylanase has been shown to generate similarly reducing-end branched structures [19]. Structure V was the only product oligosaccharide identified in this study that had a substitution on the reducing end unit. Complete structural assignment could not be made on all d.p. 3 analytes, where Structure IV was proposed as the likely structure due to the evidence that it was not substituted on the reducing end and the retention time and fragmentation pattern were not consistent with the linear structure (III). 

**Figure 6:**
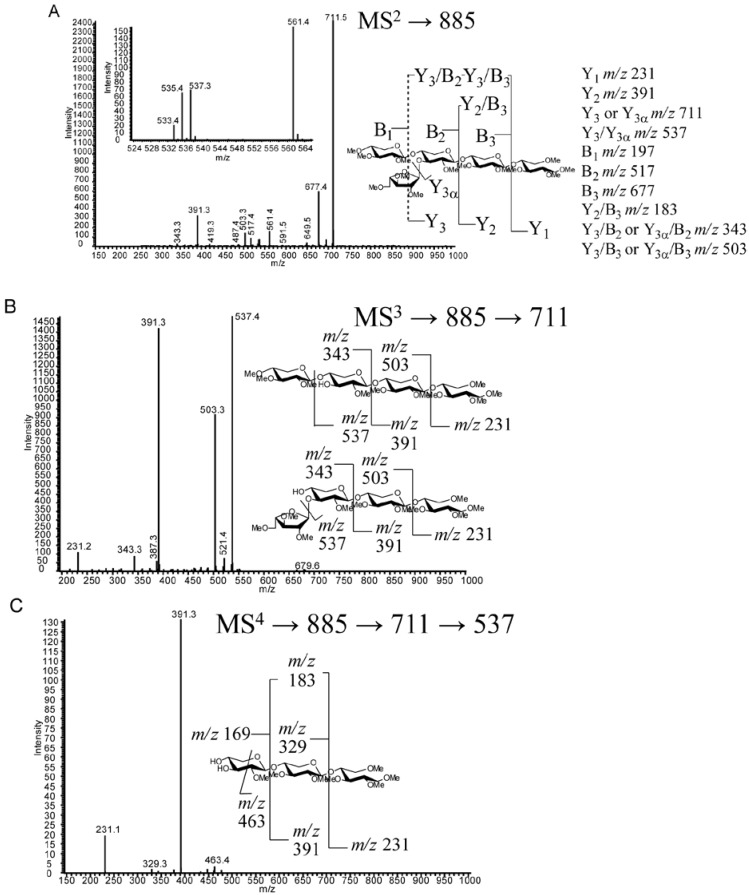
MS*^n^* fragmentation of permethylated oligosaccharide VIII (RT 28.5 min) (**a**) MS^2^* m/z* 885. (**b**) MS^3^ fragmentation *m/z* 885→711. (**c**) MS^4^ fragmentation *m/z* 885→711→537.

**Figure 7:**
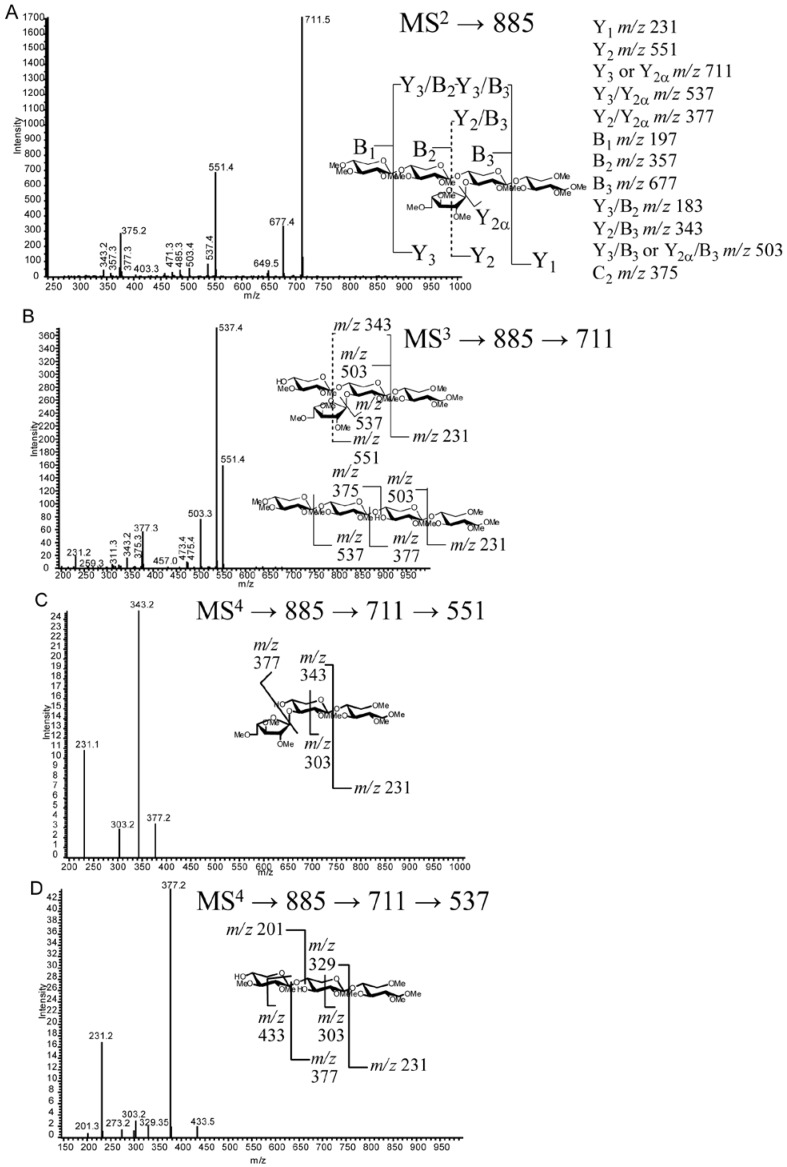
MS*^n^* fragmentation of permethylated oligosaccharide XVII (RT 27.5 min) (**a**) MS^2^* m/z* 885. (**b**) MS^3^ fragmentation *m/z* 885→711. (**c**) MS^4^ fragmentation *m/z* 885→711→551. (**d**) MS^4^ fragmentation *m/z* 885→711→537.

Longer oligosaccharides provide less ambiguous spectra due to the length of the oligosaccharide backbone, permitting more targeted cycles of fragmentation of glycosidic fragment ions. Structure VI can be determined by a combination of MS^2^ fragmentations (described in Section 2.4.), indicating the location of branching on the chain, followed by MS^3 ^and MS^4^. MS^3^ (*m/z* 725→551, Figure 5b) yields an *m/z* 377 product ion that would be the same from either fragmentation pathway consistent with the two potential fragmentation pathways of the proposed structure. The MS^4^ spectrum (*m/z* 725→551→377, Figure 5c) gives an *m/z* 231 ion indicating an unsubstituted reducing end structure. In addition, lower abundance, but structurally informative cross-ring cleavages showed the linkage site of the branching substitution (^1,4^X to give *m/z* 273.2, ^0,2^X to give *m/z* 287.2, ^2,5^X to give *m/z* 303.4). The lower energy fragmentation of the linear ion-trap (LTQ) requires the use of multiple stages of fragmentation to obtain strong cross-ring cleavages that show the linkage position of the branch; this is in contrast to the generation of diagnostic ions for arabinose branching in the positive ion tandem mass spectrum using high energy CID MALDI-TOF/TOF for the analysis of reducing-end labeled AXOs [22,23]. Each enzymatic condition resulted in only one d.p.5 peak (VIII and XVII) having different retention times and likely different structures. Structure VIII from *T. viride* endo-xylanase digestion can be assigned through a sequential fragmentation of the glycosidic bond cleavage fragment ions (to MS^5^) to locate the site of branching on the third backbone residue from the reducing end and the linkage information in the MS^4^ spectrum (Figure 6 a–c). Similarly, Structure XVII from the *T. reesei* enzyme cocktail digestion can be assigned based on the same MS*^n^* tree, but the differing spectra allow for the determination of the branch point on the second unit from the reducing end (Figure 7a–c). Structure XVII is also consistent with a structure identified and purified from a SG biomass fermentation using *T. reseei*-based enzyme cocktails [27]. MS^5^ spectra of VIII and XVII (Figure 6 and Figure 7, *m/z* of 391 and 377, respectively) show only an *m/z* 231 ion corresponding to an unsubstituted reducing end of the oligosaccharide ion. 

The combination of LC-MS, MS^2^, and MS*^n^* data provided diagnostic ions for the identification of twelve branched oligosaccharide structures (Figure 3 and Figure 4) in addition to the assignment of xylobiose and xylotriose from *T. viride* endo-xylanase digestion and nine branched product oligomers (five differing from *T. viride* endo-xylanase digestion) from *T. reesei* enzyme cocktail digestion. The use of the two different enzyme conditions demonstrates that the use of LC-MS*^n^* is capable of differentiating isomeric structures that vary only in their position and quantity of branch points on the xylose backbone. 

### 2.6. Discussion

Arabinoxylans can be conveniently depolymerized to manageable sized units by treatment with xylanase. However new methods are needed to analyze the resulting AXOs for structure because those reported in the past are laborious and insensitive to low-abundance species. The use of C_18_-LC-MS*^n^* under a relatively wide elution gradient and online MS*^n^* monitoring allowed the separation and analysis of AXOs with d.p. 3–9 with good resolution of peaks in a convenient manner. Due to the improved resolution of RP-LC-MS, low abundance structures that would be difficult to separate in sufficient quantities for full structural characterization can now be deduced. This new method has direct applications to researching biochemical conversion of biomass into biofuels. Developing minimal enzyme mixtures for converting xylan into fermentable monosaccharides is challenging because of the complexity of the xylan chains [6,7,47]. This method to identify the unhydrolyzed oligosaccharides of arabinoxylan after enzyme treatment will allow for a better understanding of the substrate selectivity of currently marketed biomass saccharifying enzymes and can be used to screen for auxiliary enzymes beneficial to saccharification. The use of permethylation is also amenable to alternate chromatography techniques (e.g., porous graphitic carbon (PGC)) that could provide further benefits in chromatographic resolution. Examples might include its use for more complex mixtures such as pericarp xylan for food applications or whole biomass digestions that contain oligosaccharides derived from the diverse carbohydrate biopolymers present within the cell wall. 

In this study, RP-LC-MS*^n^* allowed for the differentiation of multiple components from SG xylanase digestion mixtures. LC-MS allowed for the analysis of small components and separated AXOs with higher resolution than other chromatographic techniques. In comparison to another report of SG xylan characterization, xylan extracted from ball-milled Alamo variety SG was *T. viride* endo-xylanase-digested followed by fractionation by size-exclusion chromatography; the report characterized three arabinose-containing xylooligosaccharides (XO) structures of d.p 8, d.p. 6, and d.p.5, while leaving a low-mass fraction uncharacterized [20]. In this LC-MS*^n^* study, the previously reported structures from *T. viride* endo-xylanase-digested SG xylan are consistent with d.p. 5 (VIII) and d.p. 6 (X); however the previously reported d.p. 8 was not observed. In contrast to the previous report, this method identifies three d.p. 6 oligomers (IX, X, XI) as well as two oligosaccharides that could be components of the previously reported d.p.8 (VIII + III). This study used Cave-in-Rock variety SG, where structural variation could be attributed to inherent differences between the two varieties of SG (lowland octoploid Alamo *vs.* upland tetraploid Cave-in-Rock) or differences in enzymatic treatment conditions (*i.e.*, temperature, enzyme loading, and buffer salts). In addition, the use of LC-MS*^n^* makes it unnecessary to purify each component of the digestion prior to characterization, leading to the identification of several components of lower d.p. (≤4) than were previously reported for SG xylan [20]. 

One area not previously reported on is the effect of harvest maturity on the xylan structure. SG xylan fractions A and B were isolated from three harvest maturities. Interestingly, the structures obtained from the endpoint enzymatic depolymerization of SG xylan A and B did not change with maturity. While the diversity of AXO structures remained uniform with plant development, there are differences in the relative occurrence of sequences as SG ages. This result bodes well for biorefineries because it suggests that an enzyme mixture formulated with the activities necessary to hydrolyze all SG xylan glycosidic bonds should be effective regardless of the harvest maturity of the biomass delivered to the gate. 

While this is the first structure analysis that has considered SG maturity as a variable, caution must be taken in drawing conclusions from the use of endpoint enzyme-based characterization of xylan, as substrate specificity of enzymes may shape the patterns observed, thereby creating a subset of analytes that may not fully reflect the diversity of the entire xylan chain (*i.e.* arrangement of the detected end-point oligosaccharides within the structure). Specifically, end-point digestions of *T. viride* endo-xylanase released xylobiose and *T. reesei* enzyme cocktail released both xylose and arabinose, thereby making extended structural determination impossible; therefore, less stringent digestion conditions (e.g., 50% digestion) would provide a distribution of oligosaccharides that could provide further structural information about the structural repeats of xylan. 

It should be also noted that the methods used (e.g., alkali extraction and permethylation) would hydrolyze ester bonds connected to the xylan structure (e.g., acetyl- and feruloyl-) that may vary during the maturation of SG biomass. The degree of esterification may play a role in the varied response to acidic conditions; however, ammonia-based pretreatments should remove the majority of the ester groups [27,48]. Finally, this report used whole ground plant material because that is what will be delivered to a biorefinery facility. Grass stems grow upwards by adding internodes of younger maturity. It is possible that fractionation of stems into individual internodes would yield differences hidden by using bulk samples. Future studies will focus on the use of xylan extraction techniques that retain ester functionalities, investigation of samples fractionated by internodes, and samples processed under less stringent hydrolysis conditions. 

The use of LC-MS*^n^* to identify the structural characteristics of xylan can be an important tool for biomass research due to the highly complex substrates proposed for conversion. While NMR will remain an essential technique for absolute structural identification of purified products, the sensitivity of mass spectrometry allows for the detection of many enzymatic products that are of low abundance and would be difficult to obtain in sufficient quantities for traditional characterization. The influence of low abundance structures in bioconversion is yet to be determined; however, the need for high solid loadings required for economical biomass conversion processes means the quantity of unhydrolyzed xylan structures will be in greater abundance than the enzymes potentially impeding the depolymerization. 

## 3. Experimental Section

### 3.1. Materials

Sodium chlorite, potassium hydroxide (KOH), 1-octanol, sodium hydroxide (NaOH) (50% w/v), sodium acetate anhydrous, trifluoroacetic acid (TFA), acetonitrile (ACN), acetic acid (AcOH), dimethylsulfoxide (DMSO), methyl iodide, sodium hydroxide powder, sodium borohydride, xylose, and arabinose were purchased from Sigma-Aldrich Company (St. Louis; MO). Acetone was purchased from Fisher Scientific (Pittsburgh, PA). Formic acid was purchased from Fluka Chemical (Buchs, Switzerland). Purified xylobiose to xylohexaose standards, wheat arabinoxylan, and xylanase M1 from *T. viride* were purchased from Megazyme (International Ireland Ltd (Wicklow,Ireland). Celluclast 1.5 L (Novozymes) was purchased from Brenntag Great Lakes, LLC. 

### 3.2. Xylan Sample Preparation

*Delignification and xylan isolation*. Triplicate samples of switchgrass (SG) Cave-in-Rock variety at three stages (pre-boot, anthesis, and post frost) of maturity were delignified and the xylan was alkali extracted as previously described [28]. The compositions of holocellulose and xylan components were quantified using two-stage acid hydrolysis (NREL procedure LAP002) [33], as previously described [28]. 

*T. viride xylanaseDigestion*. Triplicate samples (normalized for xylose content to 1 mg of MPV-1 xylan A) of each SG xylan maturity were exhaustively digested; in 50 mM sodium acetate pH 5.0 (1 mL); by the action of 1 μL of xylanase from *T. viride* (2000 U/mL; final loading (U/mL): 2 U xylanase activity; 7.8 μg protein) solution was added in two portions 3 h apart, with a total reaction time of 16 h at 50 °C. 

*CelluclastDigestion*. Triplicate samples (1 mg) of each SG xylan maturity were exhaustively digested, in 50 mM sodium acetate pH 5.0 (1 mL), by the action of 2 μL of Celluclast 1.5 (solution was added in two portions 3 h apart (final loading (U/mL): 2.2 U xylanase activity; 0.032 filter paper units (FPU); 1.9 U carboxymethyl cellusase activity; 0.030 U β-glucosidase; 0.012 U β-xylosidase; 0.062 U α-L- arabinofuranosidase; 30 μg protein, assayed as described in [41]), with a total reaction time of 16 h at 50 °C. 

*Analysis by HPAEC-PAD.* Twenty-five microliters of each sample (2.5 μg) were analyzed by HPAEC-PAD (Dionex ACS 3000, Sunnyvale, CA) utilizing a PA-100 column (Dionex) at 1 mL/min running 100% A (A:100 mM NaOH) isocratically for 15 min followed by a gradient program to 12% B (B:100 mM NaOH containing 1 M sodium acetate) over 20 min followed by washout and 15 min of re-equilibration in 100% A, based on conditions reported in [15]. Extent of digestion was determined by comparison of xylose, arabinose, and linear xylooligosaccharides (xyl_2_-xyl_6_) released by enzymatic hydrolysis *versus* the amount of xylose and arabinose produced by acid hydrolysis (2M TFA) of equivalent portion of xylan. 

### 3.3. Permethylation Reaction of Enzymatic Products

An aliquot of each digestion (100 μg) was removed, maltotetraose was added as an internal standard, and dried in a Speed vac. After drying, samples were reduced with sodium borohydride (500 mM) in 2 M NH_4_OH for 1 h at 45 °C. Excess borohydride was removed by repeated addition of acidic methanol and dried by speed vac (3×). Permethylation (PM) was performed according to the method of Ciucanu and Kerek [49], with modifications to limit oxidative degradation [50]. Due to the presence of undermethylated material (<5%) in preliminary experiments, each sample was methylated twice to ensure the reaction was complete. Samples were dissolved in 300 μL methanol followed by addition of 700 μL water. 

### 3.4. C18-LC-MS^n^.

*LC-MS*. Mass spectrometry samples (2 μL injection, 0.2 μg based on starting material) were analyzed by LC-MS (Thermo Acella HPLC) through a narrow-bore (2.1 mm × 250 mm, 3 μm particle size) C18 column (Inertsil, GL Sciences, Inc., Torrance, CA) running a gradient elution of 30% A:70% B (buffer A 0.1% formic acid, buffer B 100% acetonitrile) to 70% A:30% B over 45 min at a flow rate of 200 μL/min, followed by a 5 min B washout and 10 min re-equilibration, while maintaining a constant column temperature of 30 °C. Electrospray positive mode ionization data were collected with a linear ion trap-Orbitrap mass spectrometer (Thermo LTQ-Orbitrap Discovery) under Xcalibur 2.1 control. Prior to LC-MS*^n^* experiments the instrument was tuned and calibrated using the LTQ tune mix. The parent ion table function was used to isolate and fragment singly charged ions ([M+Na]^1+^) corresponding to permethylated-labeled arabino-xylooligosaccharides, and hexose or methyl-hexuronic acids containing xylooligosaccharides using a 2 dalton isolation window to encompass the isotopic window of each isolated ion (Table 1). The fragmentation energy (CID) was set to 55%, as this provided informative fragmentation from permethylated linear xylooligosaccharide (degree of polymerization (d.p.) 5, X_5_-PM, *m/z* 885.2 [M+Na]^1+^) and a d.p. 5-PM derived from wheat arabinoxylan as a model branched oligomer introduced by infusion. MS data was collected in both linear ion-trap mode and FT-Orbitrap mode and compiled into graphical form using Microsoft Excel, where peak areas from extracted ion chromatograms (EICs) from Orbitrap data using a 1 *m/z* window for each composition are reported. Standard deviations were calculated from values using triplicate digestion and labeling experiments. All structures were determined by *de novo* interpretation of the tandem and MS*^n^* fragmentation data.

#### MS^n^ Method

Due to the number of scans desired to maximize structural data, only linear ion-trap data was used due to the faster analysis speeds. Segment 1: 14 min; MS^2^ (245); MS^2^ (405.2); Segment 2: 6 min (14–20 min); MS^2^ 565.30; MS^3^ (565.30→391.30); MS^3^ (565.30→357.20); Segment 3: 6 min (20–26 min); MS^2^ 725.50; MS^3^ (725.50→551.50); MS^4 ^(725.50→ 551.50→377.30); MS^4 ^(725.50→ 551.50→391.30); Segment 4: 7.20 min (26-33.2min); MS^2^ (885.40); MS^3 ^(885.40→ 711.40); MS^4^ (885.40→711.40→537.40); MS^5 ^(885.40→711.40→537.40→377.30); MS^5 ^(885.40→711.40→537.40→391.30); MS^4 ^(885.40)→711.40→551.50); MS^5^ (885.40→711.40→ 551.50→377.30); Segment 5: 5 min (33.2–38.2 min); MS^2^ (1045.50); MS^3^ (1045.50→871.50); MS^4^ (1045.50→871.50→697.50); MS^5^ (1045.50→871.50→697.50→537.40); MS^6^ (1045.50→871.50→697.50→537.40→377.30); MS^4^ (1045.50→871.50→537.40); MS^3^ (1045.50→837.50); MS^4^(1045.50→837.50→677.40); MS^6 ^(1045.50→871.50→697.50→537.40→391.30); MS^3^ (1045.50→857.60); MS^4^ (1045.50→857.60→697.50); MS^5^ (1045.50→857.60→697.50→537.40); MS^6^ (1045.50→857.50→697.50→537.40→377.30); Segment 6: 5.6 min (38.2-43.8 min); MS^2^ (1205.60); MS^3^ (1205.60→1031.60); MS^4^ (1205.60→1031.60→857.60); MS^5^ (1205.60→1031.60→857.60→697.60); MS^5^ (1205.60→1031.60→857.60→711.40); Segment 7: 10.2 min (43.8–54.0 min); MS^2^ (1365.60); MS^3^ (1365.60→1191.70); MS^4^ (1365.60→1191.70→1031.60); MS^5^ (1365.60→1191.70→1031.60→871.50); MS^6^ (1365.60→1191.70→1031.60→871.50→711.40); Segment 8: 11 min (54–65 min)-MS only. 

## 4. Conclusions

Arabino-xylooligomers derived from switchgrass xylan of three maturities were characterized by LC-MS*^n^*. The presence of 19 saccharide products of endo-xylanase digestion by two different enzymatic treatments could be discerned by backbone residue location of branches by MS^2^ and point of attachment by MS*^n^*. The two enzymatic conditions provided different sets of product oligosaccharides due to different enzymatic substrate specificities. There are detectable differences in the quantities of xylose-normalized xylan oligosaccharides released by endo-xylanase activity with maturity, consistent with the measurements of increasing xylose:arabinose ratio of each xylan component. The variations in oligosaccharide content are different between xylan A and xylan B chains, where xylan A has a more consistent xylose:arabinose ratio of substitution with maturity, whereas xylan B has a decreasing amount of arabinose (*i.e.*, it is less substituted with maturity). The observation that the xylan structure appears uniform with maturity is beneficial showing that enzyme cocktails capable of hydrolyzing xylan glycosidic bonds need not be altered depending upon harvest maturity of the SG biomass; however, the quantity of xylan may be a factor for the loading of enzyme cocktail to limit product inhibition of enzymes. The application of permethylation followed by LC-MS*^n^* provides a useful platform for the identification of structural features of heteroxylan-derived oligosaccharides for applications to extended structure determination and sampling of biomass-derived fermentation residues to identify recalcitrant structures. 

## Supplementary Files

Supplementary File 1 PDF-Document (PDF, 1206 KB)
