# Supplementary material for: Comparative Analysis of End Point Enzymatic Digests of Arabino-Xylan Isolated from Switchgrass (Panicum virgatum L) of Varying Maturities using LC-MSn †
Source: Metabolites. 2012 Nov 19;2(4):959–82. doi: 10.3390/metabo2040959 (PMC3901237; doi:10.3390/metabo2040959)

Figure S1. HPAEC-PAD of end-point enzymatic digestions (**a**) stacked chromatogram of MPV-1, MPV-2, and MPV-3 xylan A digested with *T. viride* endo-xylanase (**b**) stacked chromatogram of MPV-1, MPV-2, and MPV-3 xylan A digested with Celluclast. Graphical representation of SG xylan A digestion with (**c**) *T. viride* endo-xylanase and (**d**) Celluclast.

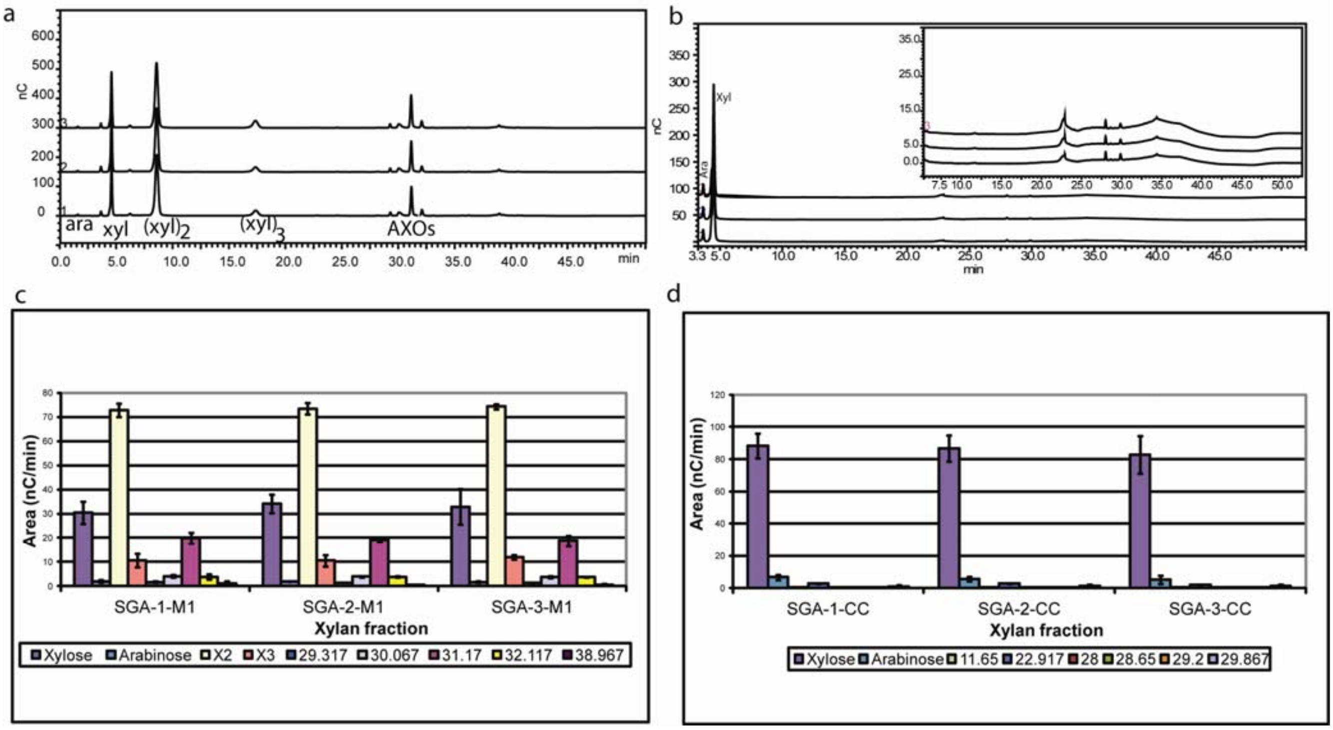

Figure S2. Normalized C_{18}-LC-MS of enzymatically depolymerized MPV-2 xylan B (**a**) Oligosaccharide products of *T. viride* endo-xylanase enzymatic end point treatment of switchgrass xylan. (**b**) Oligosaccharide products of Celluclast end point treatment of switchgrass xylan. EICs corresponding to: brown $(Pent)_2$ PM (*m/z* 405); green $(Pent)_3$ PM (*m/z* 535); blue $(Pent)_4$ PM (*m/z* 725); gold $(Pent)_5$ PM (*m/z* 885); purple $(Pent)_6$ PM (*m/z* 1045); aqua $(Pent)_7$ PM (*m/z* 1205); grey $(Pent)_8$ PM (*m/z* 1365).

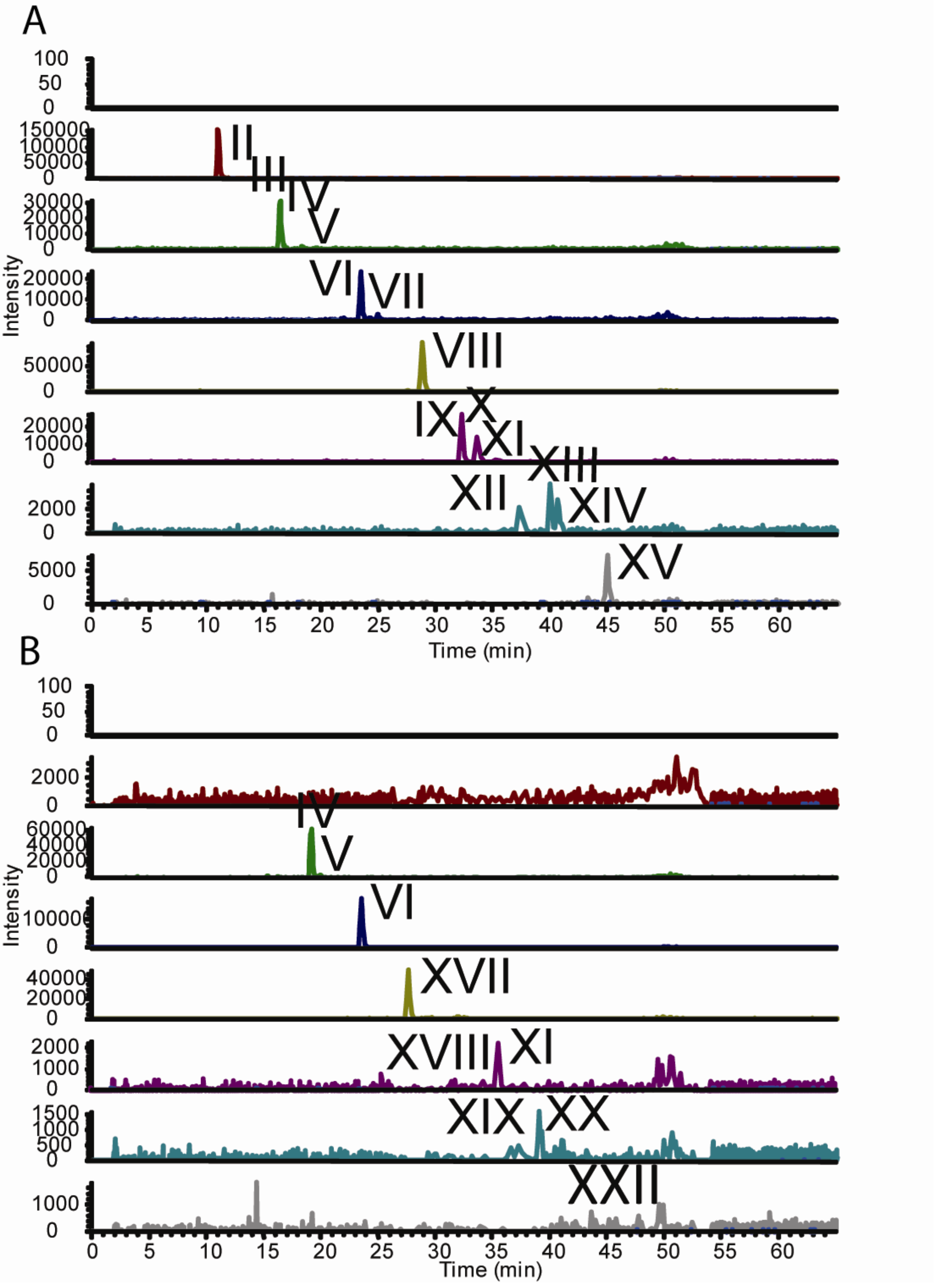

Figure S3. MS^n fragmentation of permethylated oligosaccharide V (RT 19.8 min) (**a**) MS^2 *m/z* 565. (**b**) MS^3 fragmentation *m/z* 565→391.

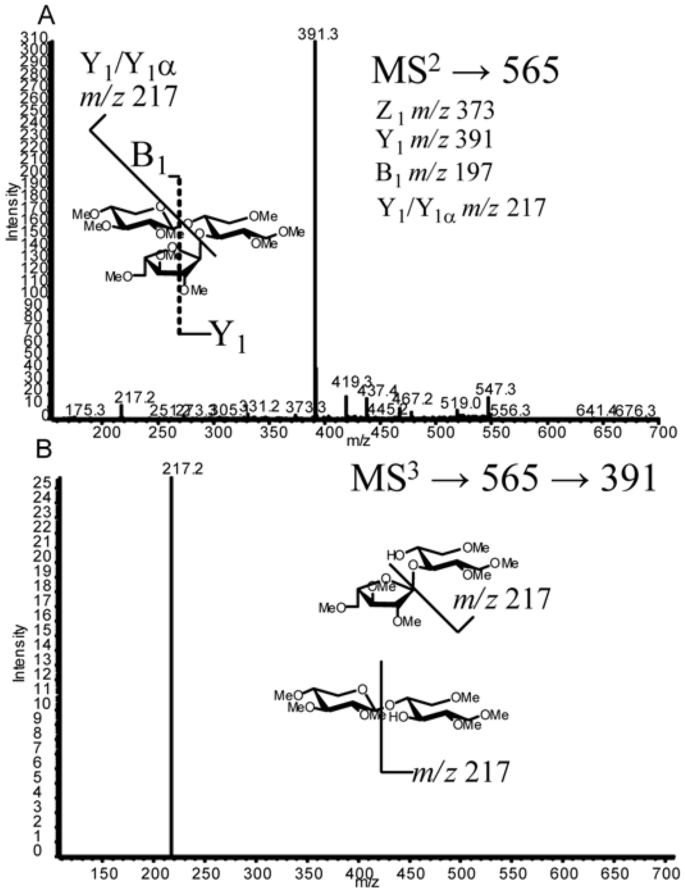

Figure S4. MS^n fragmentation of permethylated oligosaccharide IX (RT 32.4 min) (**a**) MS^2 *m/z* 1045. (**b**) MS^3 fragmentation *m/z* 1045→871. (**c**) MS^4 fragmentation *m/z* 1045→871→697.

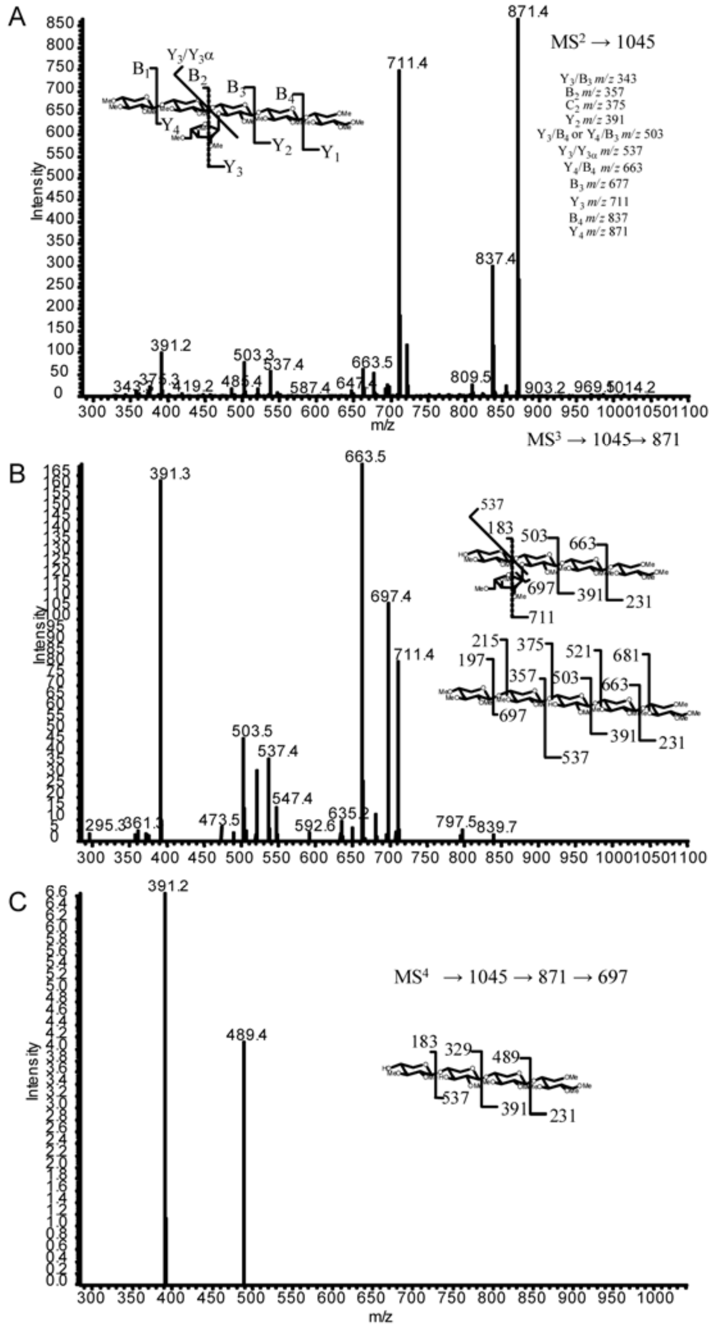

Figure S5. MS^n fragmentation of permethylated oligosaccharide X (RT 33.6 min) (**a**) MS^2 *m/z* 1045. (**b**) MS^3 fragmentation *m/z* 1045→871. (**c**) MS^4 fragmentation *m/z* 1045→871→697.

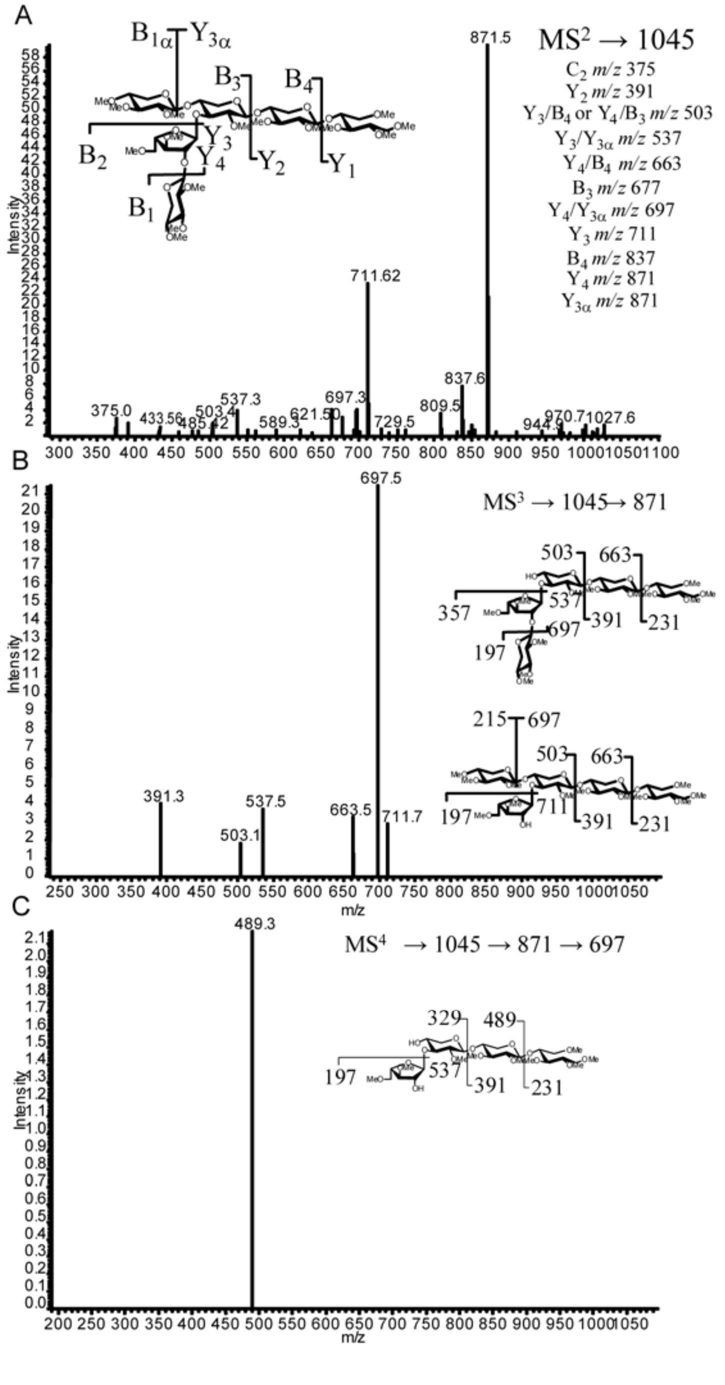

Figure S6. MS^n fragmentation of permethylated oligosaccharide XI (RT 35.5 min) (**a**) MS^2 *m/z* 1045. (**b**) MS^3 fragmentation *m/z* 1045→871. (**c**) MS^4 fragmentation *m/z* 1045→871→697.

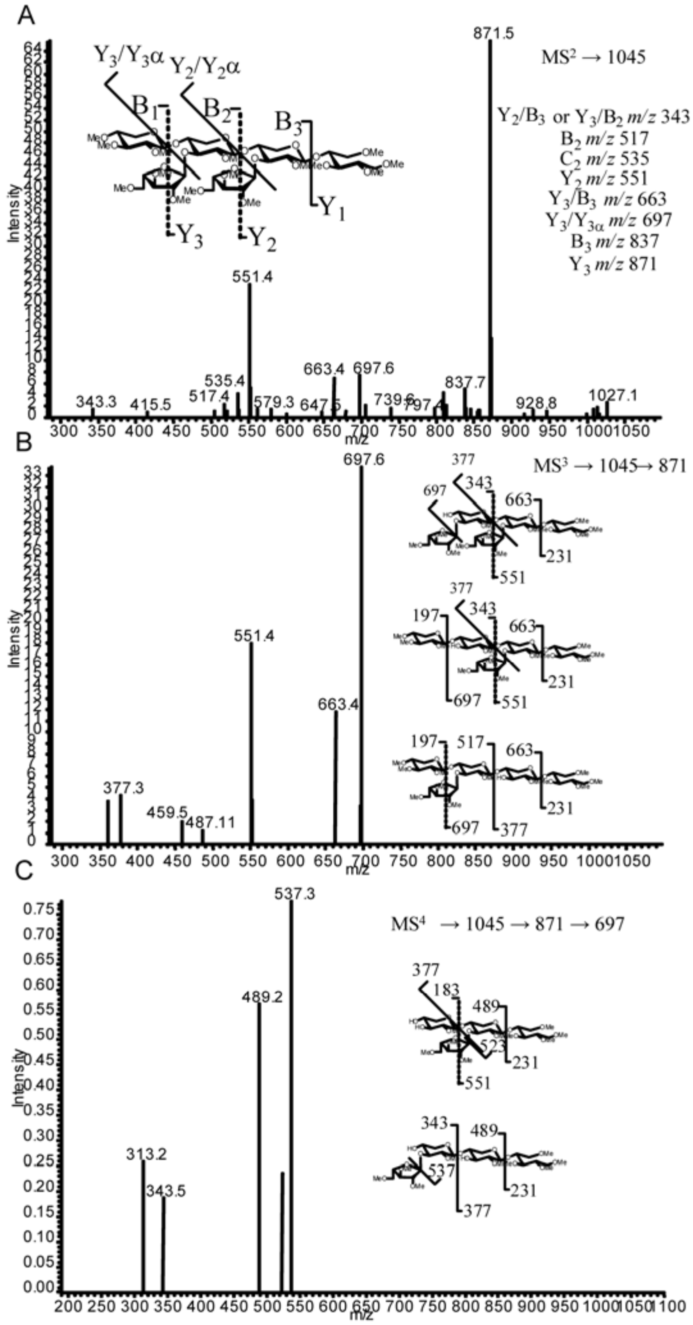

Figure S7. MS^n fragmentation of permethylated oligosaccharide XII (RT 37.5 min) (**a**) MS^2 *m/z* 1205. (**b**) MS^3 fragmentation *m/z* 1205→1031. (**c**) MS^4 fragmentation *m/z* 1205→1031→857. (**d**) MS^5 fragmentation *m/z* 1205→1031→857→697.

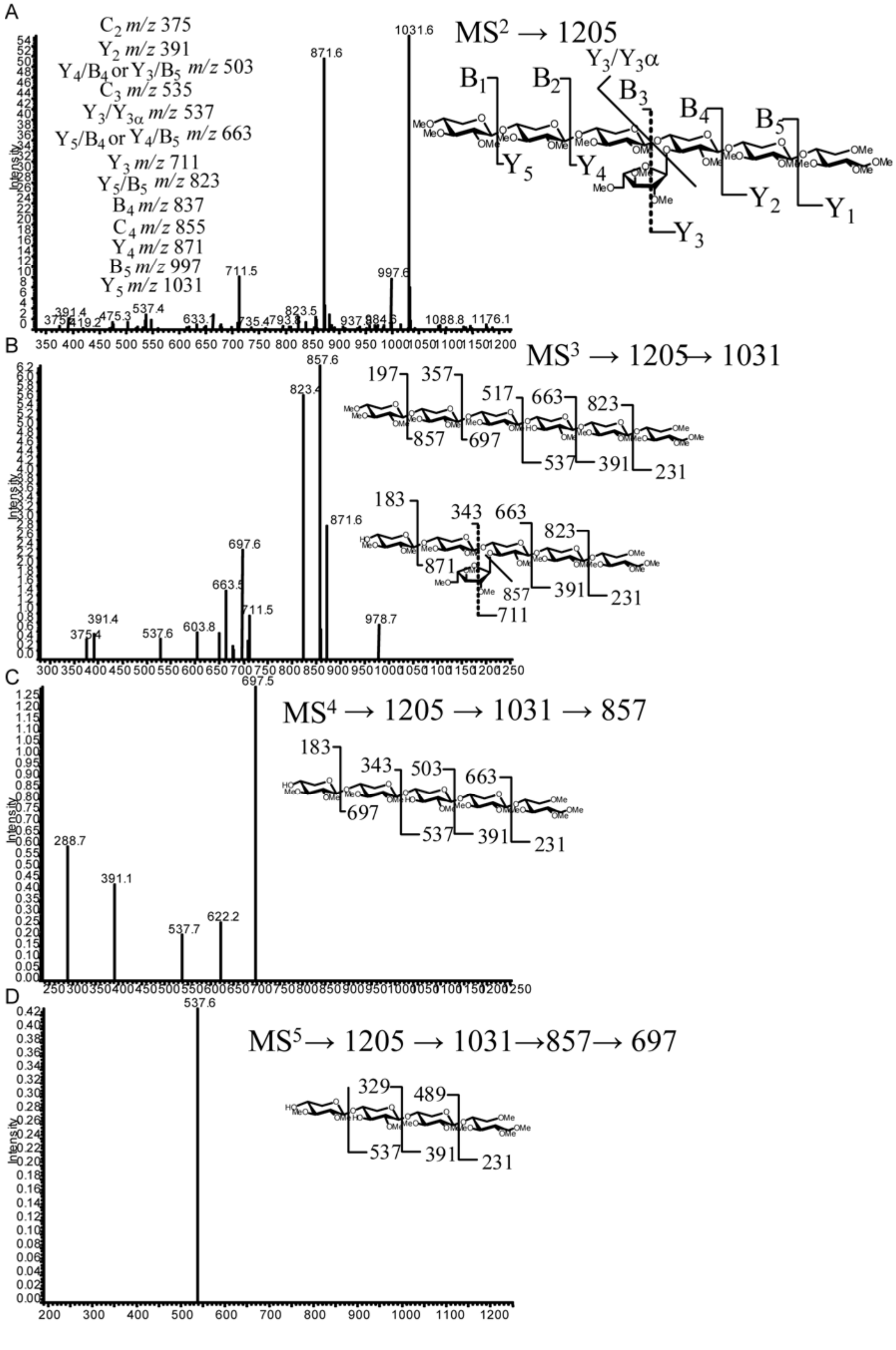

Figure S8. MS^n fragmentation of permethylated oligosaccharide XIII (RT 39.9 min) (**a**) MS^2 *m/z* 1205. (**b**) MS^3 fragmentation *m/z* 1205→1031. (**c**) MS^4 fragmentation *m/z* 1205→1031→857. (**d**) MS^4 fragmentation *m/z* 1205→1031→857→711.

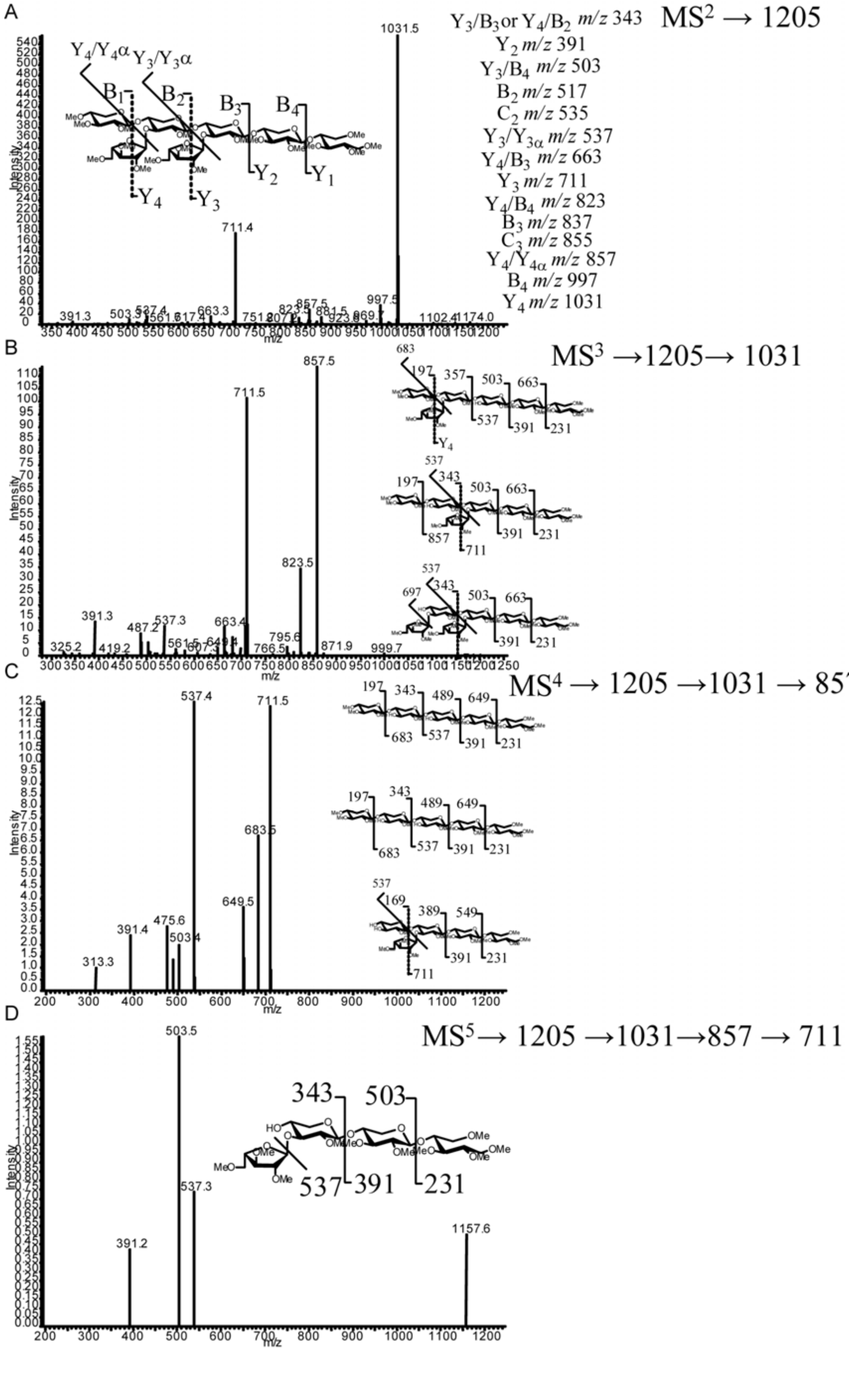

Figure S9. MS^n fragmentation of permethylated oligosaccharide XIV (RT 40.7 min) (**a**) MS^2 *m/z* 1205. (**b**) MS^3 fragmentation *m/z* 1205→1031. (**c**) MS^4 fragmentation *m/z* 1205→1031→857. (**d**) MS^4 fragmentation *m/z* 1205→1031→857→711. (

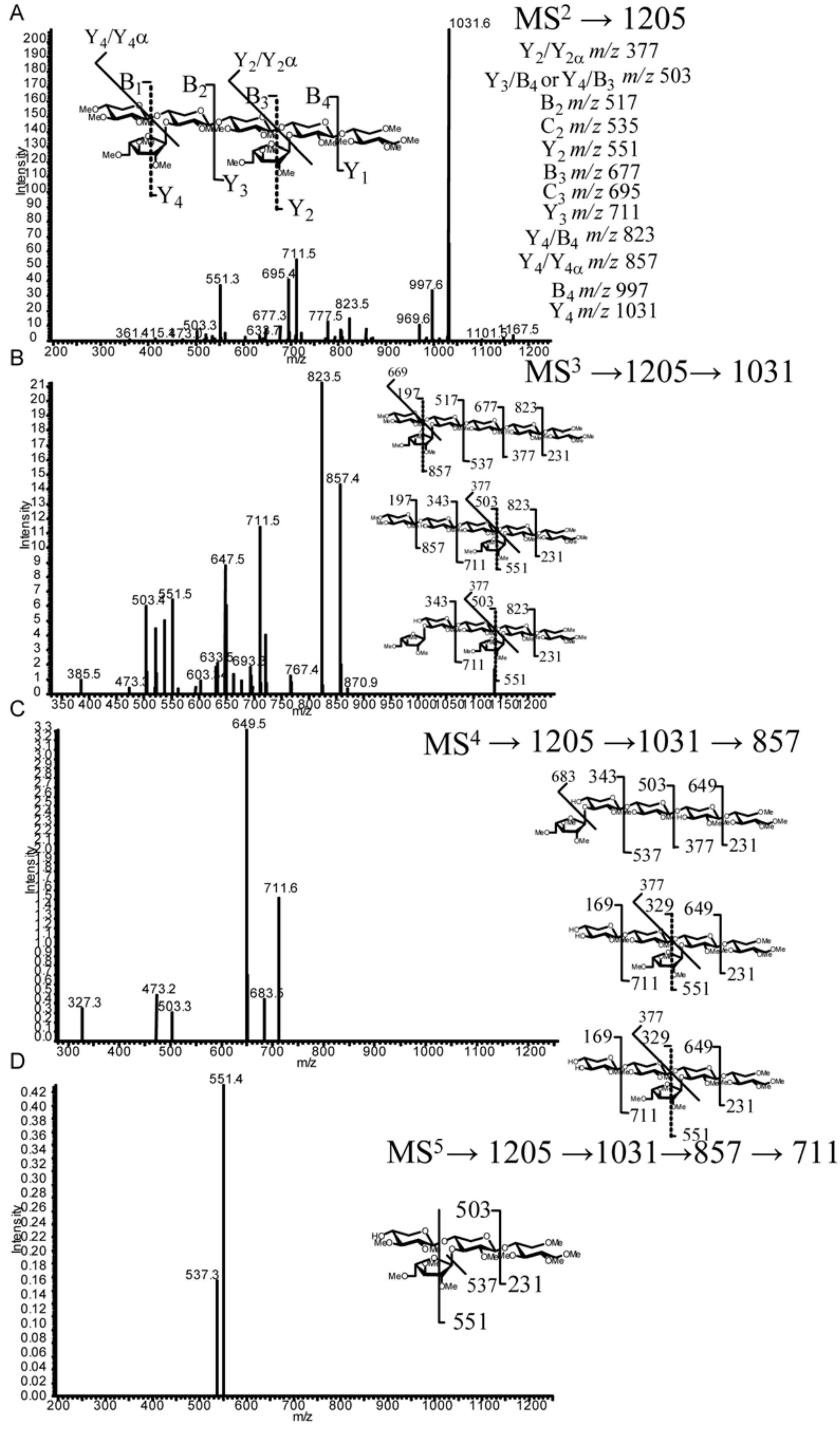

Figure S10. MS^n fragmentation of permethylated oligosaccharide XVIII (RT 32.8 min) (**a**) MS^2 *m/z* 1045. (**b**) MS^3 fragmentation *m/z* 1045→871. (**c**) MS^4 fragmentation *m/z* 1045→871→697. (**d**) MS^4 fragmentation *m/z* 1045→871→697→537.

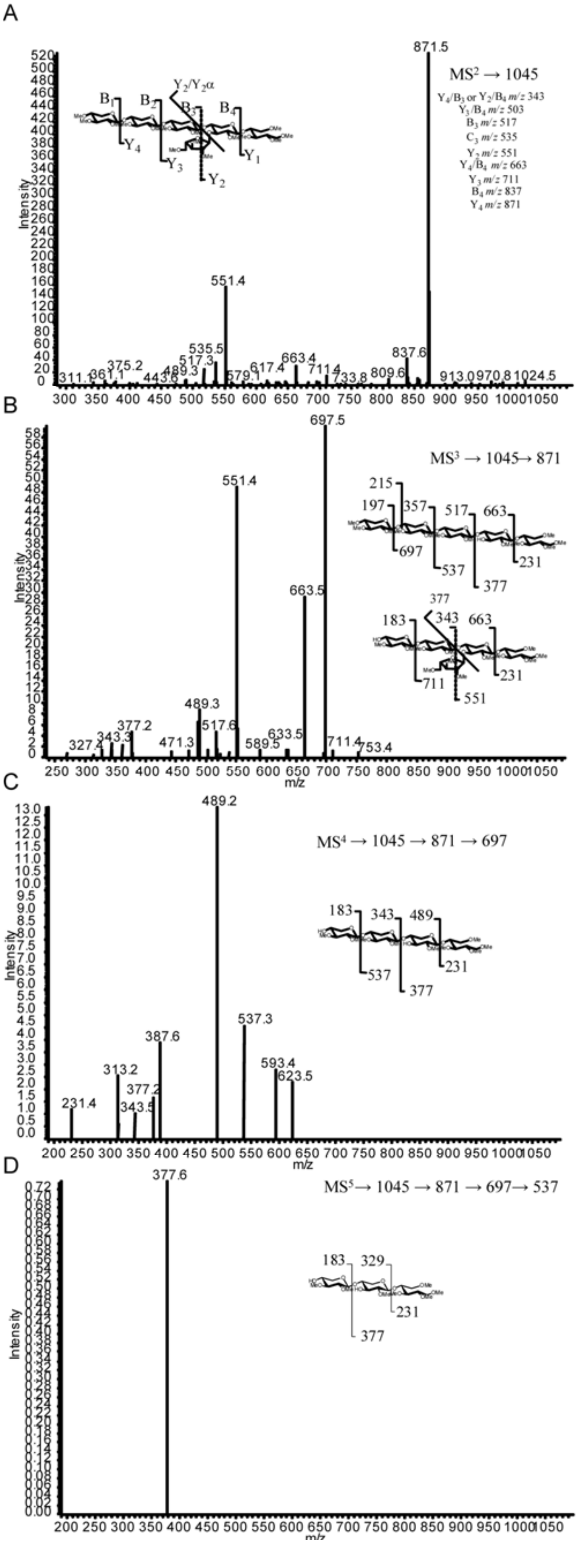

Figure S11. MS^n fragmentation of permethylated oligosaccharide XIX (RT 38.9min) (**a**) MS^2 *m/z* 1045. (**b**) MS^3 fragmentation *m/z* 1045→871. (**c**) MS^4 fragmentation *m/z* 1045→871→697. (**d**) MS^4 fragmentation *m/z* 1045→871→697→537.

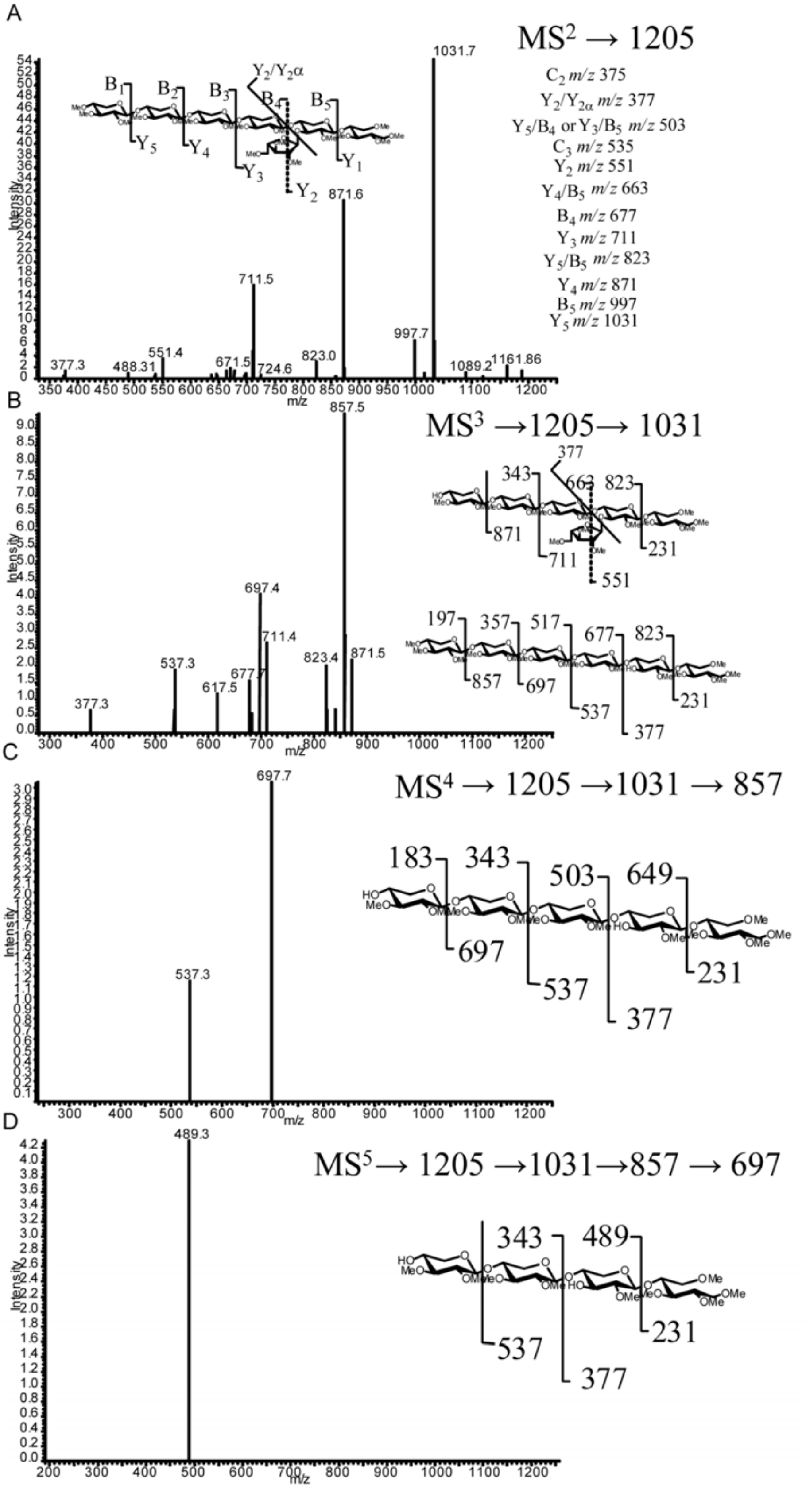

Figure S12. MS^n fragmentation of permethylated oligosaccharide XXI (RT 42.9min) (**a**) MS^2 *m/z* 1205. (**b**) MS^3 fragmentation *m/z* 1205→1031. (**c**) MS^4 fragmentation *m/z* 1205→1031→857. (**d**) MS^4 fragmentation *m/z* 1205→1031→857→697.

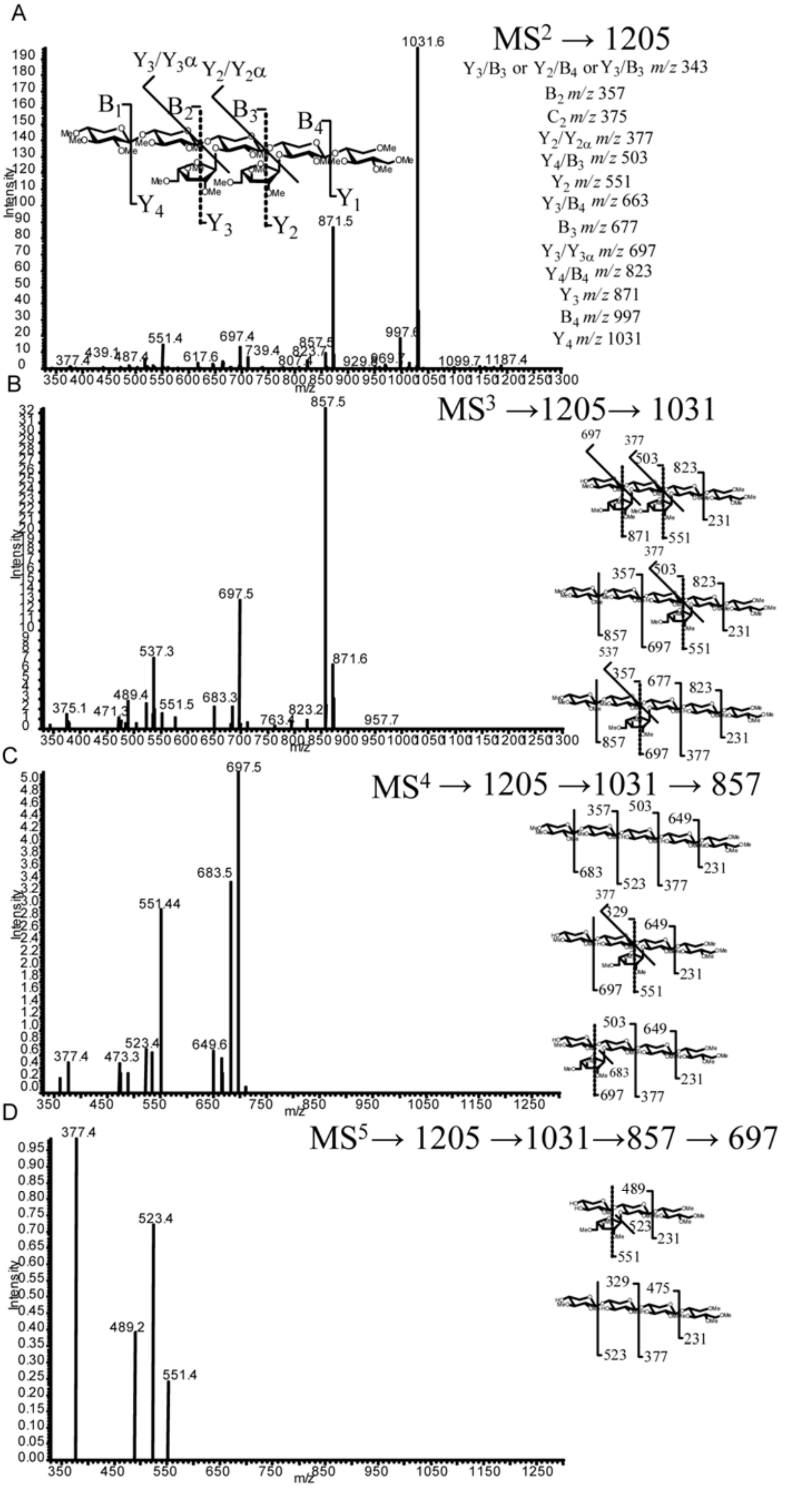

Supplement: Supplementary File 1 — PDF-Document (PDF, 1206 KB) [file metabolites-02-00959-s001.pdf]
